# Systematic Evaluation of Pleiotropy Identifies 6 Further Loci Associated With Coronary Artery Disease

**DOI:** 10.1016/j.jacc.2016.11.056

**Published:** 2017-02-21

**Authors:** Thomas R. Webb, Jeanette Erdmann, Kathleen E. Stirrups, Nathan O. Stitziel, Nicholas G.D. Masca, Henning Jansen, Stavroula Kanoni, Christopher P. Nelson, Paola G. Ferrario, Inke R. König, John D. Eicher, Andrew D. Johnson, Stephen E. Hamby, Christer Betsholtz, Arno Ruusalepp, Oscar Franzén, Eric E. Schadt, Johan L.M. Björkegren, Peter E. Weeke, Paul L. Auer, Ursula M. Schick, Yingchang Lu, He Zhang, Marie-Pierre Dube, Anuj Goel, Martin Farrall, Gina M. Peloso, Hong-Hee Won, Ron Do, Erik van Iperen, Jochen Kruppa, Anubha Mahajan, Robert A. Scott, Christina Willenborg, Peter S. Braund, Julian C. van Capelleveen, Alex S.F. Doney, Louise A. Donnelly, Rosanna Asselta, Pier A. Merlini, Stefano Duga, Nicola Marziliano, Josh C. Denny, Christian Shaffer, Nour Eddine El-Mokhtari, Andre Franke, Stefanie Heilmann, Christian Hengstenberg, Per Hoffmann, Oddgeir L. Holmen, Kristian Hveem, Jan-Håkan Jansson, Karl-Heinz Jöckel, Thorsten Kessler, Jennifer Kriebel, Karl L. Laugwitz, Eirini Marouli, Nicola Martinelli, Mark I. McCarthy, Natalie R. Van Zuydam, Christa Meisinger, Tõnu Esko, Evelin Mihailov, Stefan A. Escher, Maris Alver, Susanne Moebus, Andrew D. Morris, Jarma Virtamo, Majid Nikpay, Oliviero Olivieri, Sylvie Provost, Alaa AlQarawi, Neil R. Robertson, Karen O. Akinsansya, Dermot F. Reilly, Thomas F. Vogt, Wu Yin, Folkert W. Asselbergs, Charles Kooperberg, Rebecca D. Jackson, Eli Stahl, Martina Müller-Nurasyid, Konstantin Strauch, Tibor V. Varga, Melanie Waldenberger, Wellcome Trust Case Control Consortium, Lingyao Zeng, Rajiv Chowdhury, Veikko Salomaa, Ian Ford, J. Wouter Jukema, Philippe Amouyel, Jukka Kontto, MORGAM Investigators, Børge G. Nordestgaard, Jean Ferrières, Danish Saleheen, Naveed Sattar, Praveen Surendran, Aline Wagner, Robin Young, Joanna M.M. Howson, Adam S. Butterworth, John Danesh, Diego Ardissino, Erwin P. Bottinger, Raimund Erbel, Paul W. Franks, Domenico Girelli, Alistair S. Hall, G. Kees Hovingh, Adnan Kastrati, Wolfgang Lieb, Thomas Meitinger, William E. Kraus, Svati H. Shah, Ruth McPherson, Marju Orho-Melander, Olle Melander, Andres Metspalu, Colin N.A. Palmer, Annette Peters, Daniel J. Rader, Muredach P. Reilly, Ruth J.F. Loos, Alex P. Reiner, Dan M. Roden, Jean-Claude Tardif, John R. Thompson, Nicholas J. Wareham, Hugh Watkins, Cristen J. Willer, Nilesh J. Samani, Heribert Schunkert, Panos Deloukas, Sekar Kathiresan

**Affiliations:** a Department of Cardiovascular Sciences, University of Leicester, Leicester, United Kingdom; b NIHR Leicester Cardiovascular Biomedical Research Unit, Glenfield Hospital, Leicester, United Kingdom; c Institute for Cardiogenetics, University of Lübeck, Lübeck, Germany; d DZHK (German Research Centre for Cardiovascular Research), partner site Hamburg/Lübeck/Kiel, Lübeck, Germany; e University Heart Center Luebeck, Lübeck, Germany; f William Harvey Research Institute, Barts and The London School of Medicine and Dentistry, Queen Mary University of London, London, United Kingdom; g Department of Haematology, University of Cambridge, Cambridge, United Kingdom; h Cardiovascular Division, Department of Medicine, Washington University School of Medicine, Saint Louis, Missouri; i Department of Genetics, Washington University School of Medicine, Saint Louis, Missouri; j McDonnell Genome Institute, Washington University School of Medicine, Saint Louis, Missouri; k Deutsches Herzzentrum München, Technische Universität München, München, Germany; l DZHK, Partner Site Munich Heart Alliance, Munich, Germany; m Institut für Medizinische Biometrie und Statistik, Universität zu Lübeck, Lübeck, Germany; n Center for Population Studies, National Heart, Lung, and Blood Institute, The Framingham Heart Study, Framingham, Massachusetts; o Department of Immunology, Genetics and Pathology, Rudbeck Laboratory, Uppsala University, Sweden; p Department of Medical Biochemistry and Biophysics, Vascular Biology Unit, Karolinska Institutet, Stockholm, Sweden; q Department of Physiology, Institute of Biomedicine and Translation Medicine, University of Tartu, Tartu, Estonia; r Department of Cardiac Surgery, Tartu University Hospital, Tartu, Estonia; s Clinical Gene Networks AB, Stockholm, Sweden; t Department of Genetics & Genomic Sciences, Institute of Genomics and Multiscale Biology, Icahn School of Medicine at Mount Sinai, New York, New York; u Department of Medicine, Vanderbilt University Medical Center, Nashville, Tennessee; v Laboratory for Molecular Cardiology, Department of Cardiology, Copenhagen University Hospital Rigshospitalet, Copenhagen, Denmark; w School of Public Heath, University of Wisconsin-Milwaukee, Milwaukee, Wisconsin; x Fred Hutchinson Cancer Research Center, Seattle, Washington; y The Charles Bronfman Institute for Personalized Medicine, The Icahn School of Medicine at Mount Sinai, New York, New York; z The Genetics of Obesity and Related Metabolic Traits Program, The Icahn School of Medicine at Mount Sinai, New York, New York; aa Department of Internal Medicine, Division of Cardiovascular Medicine, University of Michigan, Ann Arbor, Michigan; bb Université de Montréal, Faculté de médecine, Département de médecine, Montreal, Quebec, Canada; cc Montreal Heart Institute, Montreal, Quebec, Canada; dd Division of Cardiovascular Medicine, Radcliffe Department of Medicine, University of Oxford, Oxford, United Kingdom; ee Wellcome Trust Centre for Human Genetics, University of Oxford, Oxford, United Kingdom; ff Center for Human Genetic Research, Massachusetts General Hospital, Boston, Massachusetts; gg Cardiovascular Research Center, Massachusetts General Hospital, Boston, Massachusetts; hh Department of Medicine, Harvard Medical School, Boston, Massachusetts; ii Program in Medical and Population Genetics, Broad Institute, Cambridge, Massachusetts; jj Samsung Advanced Institute for Health Sciences and Technology, Sungkyunkwan University, Samsung Medical Center, Seoul, South Korea; kk The Center for Statistical Genetics, Department of Genetics and Genomic Sciences, Icahn School of Medicine at Mount Sinai, New York, New York; ll The Icahn Institute for Genomics and Multiscale Biology, Department of Genetics and Genomic Sciences, Icahn School of Medicine at Mount Sinai, New York, New York; mm The Zena and Michael A. Weiner Cardiovascular Institute, Icahn School of Medicine at Mount Sinai, New York, New York; nn Department of Biostatistics, Academic Medical Center, Amsterdam, the Netherlands; oo Institute for Animal Breeding and Genetics, University of Veterinary Medicine Hannover, Hannover, Germany; pp MRC Epidemiology Unit, Institute of Metabolic Science, Addenbrooke's Hospital, Cambridge, United Kingdom; qq Department of Vascular Medicine, Academic Medical Center, Amsterdam, the Netherlands; rr Medical Research Institute, University of Dundee, Ninewells Hospital and Medical School, Scotland, United Kingdom; ss Department of Biomedical Sciences, Humanitas University, Milan, Italy; tt Humanitas Clinical and Research Center, Milan, Italy; uu Niguarda Hospital, Milan, Italy; vv Azienda Sanitaria Locale 3 San Francesco, Nuoro, Italy 3, Nuoro, Italy; ww Department of Biomedical informatics, Vanderbilt University Medical Center, Nashville, Tennessee; xx Klinik für Kardiologie, Pneumologie und Innere Medizin, Imland Klinik Rendsburg, Rendsburg, Germany; yy Institute of Clinical Molecular Biology, Christian-Albrechts-University of Kiel, Kiel, Germany; zz Institute of Human Genetics, University of Bonn, Bonn, Germany; aaa Department of Genomics, Life & Brain Center, University of Bonn, Bonn, Germany; bbb Division of Medical Genetics, Department of Biomedicine, University of Basel, Basel, Switzerland; ccc HUNT Research Centre, Department of Public Health and General Practice, Norwegian University of Science and Technology, Levanger, Norway; ddd St. Olav Hospital, Trondheim University Hospital, Trondheim, Norway; eee Department of Medicine, Levanger Hospital, Nord-Trøndelag Health Trust, Levanger, Norway; fff Department of Public Health and Clinical Medicine, Research Unit Skellefteå, Umeå University, Sweden; ggg Institute for Medical Informatics, Biometry and Epidemiology, University Hospital Essen, Essen, Germany; hhh Research unit of Molecular Epidemiology, Helmholtz Zentrum München–German Research Center for Environmental Health, Neuherberg, Germany; iii Institute of Epidemiology II, Helmholtz Zentrum München–German Research Center for Environmental Health, Neuherberg, Germany; jjj German Center for Diabetes Research, Neuherberg, Germany; kkk Institute Medizinische Klinik und Poliklinik, Klinikum rechts der Isar der Technischen Universität München, Munich, Germany; lll Department of Medicine, Section of Internal Medicine, University of Verona, Verona, Italy; mmm Oxford Centre for Diabetes, Endocrinology and Metabolism, University of Oxford, Oxford, United Kingdom; nnn Oxford National Institute for Health Research Biomedical Research Centre, Churchill Hospital, Old Road Headington, Oxford, Oxford, United Kingdom; ooo Estonian Genome Center, University of Tartu, Tartu, Estonia; ppp Division of Endocrinology, Boston Children's Hospital, Boston, Massachusetts; qqq Department of Genetics, Harvard Medical School, Boston, Massachusetts; rrr Broad Institute of the Massachusetts Institute of Technology and Harvard University, Cambridge, Massachusetts; sss Genetic and Molecular Epidemiology Unit, Lund University Diabetes Centre, Department of Clinical Sciences, Lund University, Malmö, Sweden; ttt Institute of Molecular and Cell Biology, Tartu, Estonia; uuu School of Molecular, Genetic and Population Health Sciences, University of Edinburgh, Medical School, Teviot Place, Edinburgh, Scotland, United Kingdom; vvv National Institute for Health and Welfare (THL), Helsinki, Finland; www Ruddy Canadian Cardiovascular Genetics Centre, University of Ottawa Heart Institute, Ottawa, Ontario, Canada; xxx Princess Al-Jawhara Al-Brahim Centre of Excellence in Research of Hereditary Disorders (PACER-HD), King Abdulaziz University, Jeddah, Saudi Arabia; yyy Merck Sharp & Dohme, Rahway, New Jersey; zzz Department of Cardiology, Division Heart & Lungs, UMC Utrecht, the Netherlands; aaaa Durrer Center for Cardiogenetic Research, ICIN-Netherlands Heart Institute, Utrecht, the Netherlands; bbbb Institute of Cardiovascular Science, Faculty of Population Health Sciences, University College London, London, United Kingdom; cccc Division of Endocrinology, Diabetes and Metabolism, Department of Medicine, Ohio State University, Columbus, Ohio; dddd Department of Psychiatry, Icahn School of Medicine at Mount Sinai, New York, New York; eeee Institute of Genetic Epidemiology, Helmholtz Zentrum München–German Research Center for Environmental Health, Neuherberg, Germany; ffff Department of Medicine I, University Hospital Grosshadern, Ludwig-Maximilians-Universität, Munich, Germany; gggg Institute of Medical Informatics, Biometry and Epidemiology, Chair of Genetic Epidemiology, Ludwig-Maximilians-Universität, Munich, Germany; hhhh MRC/BHF Cardiovascular Epidemiology Unit, Department of Public Health and Primary Care, University of Cambridge, Cambridge, United Kingdom; iiii Robertson Centre for Biostatistics, University of Glasgow, Glasgow, United Kingdom; jjjj Department of Cardiology, Leiden University Medical Center, Leiden and Interuniversity Cardiology Institute of the Netherlands, Utrecht, the Netherlands; kkkk Université de Lille, Inserm, CHU Lille, Institut Pasteur de Lille, U1167–RID-AGE, Lille, France; llll Copenhagen University Hospital and Faculty of Health and Medical Sciences, University of Copenhagen, Copenhagen, Denmark; mmmm Toulouse University School of Medicine, Toulouse, France; nnnn Department of Biostatistics and Epidemiology, Perelman School of Medicine, University of Pennsylvania, Philadelphia, Pennsylvania; oooo Center for Noncommunicable Diseases, Karachi, Pakistan; pppp British Heart Foundation, Glasgow Cardiovascular Research Centre, University of Glasgow, Glasgow, United Kingdom; qqqq Department of Epîdemiology and Public Health, University of Strasbourg, Strasbourg, France; rrrr National Institute of Health Research Blood and Transplant Research Unit in Donor Health and Genomics, University of Cambridge, Cambridge, United Kingdom; ssss Wellcome Trust Sanger Institute, Hinxton, Cambridge, United Kingdom; tttt Parma University Hospital, Parma, Italy; uuuu Department of Nutrition, Harvard School of Public Health, Boston, Massachusetts; vvvv Department of Public Health & Clinical Medicine, Umeå University Hospital, Umeå, Sweden; wwww Leeds Institute of Genetics, Health and Therapeutics, University of Leeds, Leeds, United Kingdom; xxxx Institute of Epidemiology and Biobank popgen, Christian-Albrechts-University Kiel, Kiel, Germany; yyyy Institute of Human Genetics, Helmholtz Zentrum München–German Research Center for Environmental Health, Neuherberg, Germany; zzzz Institute of Human Genetics, Technische Universität München, Munich, Germany; aaaaa Duke Molecular Physiology Institute, Duke University, Durham, North Carolina; bbbbb Division of Cardiology, Department of Medicine, Duke University, Durham, North Carolina; ccccc Department of Clinical Sciences in Malmo, Lund University, Clinical Research Center, Malmo, Sweden; ddddd Department of Clinical Sciences, Diabetes and Endocrinology, Lund University, University Hospital Malmo, Malmo, Sweden; eeeee Department of Genetics, Cardiovascular Institute and Institute of Translational Medicine and Therapeutics, Perelman School of Medicine, University of Pennsylvania, Philadelphia, Pennsylvania; fffff Division of Cardiology, Department of Medicine and the Irving Institute for Clinical and Translational Research, Columbia University, New York, New York; ggggg The Mindich Child Health and Development Institute, The Icahn School of Medicine at Mount Sinai, New York, New York; hhhhh Department of Epidemiology, University of Washington, Seattle, Washington; iiiii Department of Pharmacology, Vanderbilt University Medical Center, Nashville, Tennessee; jjjjj Department of Health Sciences, University of Leicester, Leicester, United Kingdom; kkkkk Department of Computational Medicine and Bioinformatics, University of Michigan, Ann Arbor, Michigan; lllll Department of Human Genetics, University of Michigan, Ann Arbor, Michigan; mmmmm Cardiology Division, Massachusetts General Hospital, Boston, Massachusetts

**Keywords:** cholesteryl ester transfer protein, expression quantitative trait loci, genetics, genome-wide association, single nucleotide polymorphism, BMI, body mass index, CAD, coronary artery disease, CETP, cholesteryl ester transfer protein, eQTL, expression quantitative trait locus, GWAS, genome-wide association study, HDL, high-density lipoprotein, LD, linkage disequilibrium, LDL, low-density lipoprotein, SNP, single nucleotide polymorphism

## Abstract

**Background:**

Genome-wide association studies have so far identified 56 loci associated with risk of coronary artery disease (CAD). Many CAD loci show pleiotropy; that is, they are also associated with other diseases or traits.

**Objectives:**

This study sought to systematically test if genetic variants identified for non-CAD diseases/traits also associate with CAD and to undertake a comprehensive analysis of the extent of pleiotropy of all CAD loci.

**Methods:**

In discovery analyses involving 42,335 CAD cases and 78,240 control subjects we tested the association of 29,383 common (minor allele frequency >5%) single nucleotide polymorphisms available on the exome array, which included a substantial proportion of known or suspected single nucleotide polymorphisms associated with common diseases or traits as of 2011. Suggestive association signals were replicated in an additional 30,533 cases and 42,530 control subjects. To evaluate pleiotropy, we tested CAD loci for association with cardiovascular risk factors (lipid traits, blood pressure phenotypes, body mass index, diabetes, and smoking behavior), as well as with other diseases/traits through interrogation of currently available genome-wide association study catalogs.

**Results:**

We identified 6 new loci associated with CAD at genome-wide significance: on 2q37 (*KCNJ13-GIGYF2*), 6p21 (*C2*), 11p15 (*MRVI1-CTR9*), 12q13 (*LRP1*), 12q24 (*SCARB1*), and 16q13 (*CETP*). Risk allele frequencies ranged from 0.15 to 0.86, and odds ratio per copy of the risk allele ranged from 1.04 to 1.09. Of 62 new and known CAD loci, 24 (38.7%) showed statistical association with a traditional cardiovascular risk factor, with some showing multiple associations, and 29 (47%) showed associations at p < 1 × 10^−4^ with a range of other diseases/traits.

**Conclusions:**

We identified 6 loci associated with CAD at genome-wide significance. Several CAD loci show substantial pleiotropy, which may help us understand the mechanisms by which these loci affect CAD risk.

Over the past decade, genome-wide association studies (GWAS) have identified several thousand robust associations (p < 5 × 10^−8^) for a range of human traits and diseases. For coronary artery disease (CAD), 56 such loci have been identified so far, explaining ∼15% of the disease’s heritability 1, 2. Approximately one-third of the CAD loci also show association with a known or putative cardiovascular risk factor, particularly blood pressure and lipid traits (2). Furthermore, several loci show association with other diseases; for example, the CAD-associated variants in the chromosome 9p21 locus also associate with risk of stroke as well as abdominal, aortic, and intracranial aneurysms 3, 4. These observations suggest that a comprehensive analysis of variants associated with other diseases and traits might not only identify additional loci associated with risk of CAD, but also provide important insights into genetic mechanisms shared by different diseases.

Here, we leveraged the HumanExome BeadChip (Illumina, Inc., San Diego, California) to test the contribution of 29,393 common variants of single nucleotide polymorphisms (SNPs) (minor-allele frequency >5%) for association with CAD. The variants included the majority of reported trait-/disease-associated lead SNPs in the National Human Genome Research Institute GWAS catalogue as of August 2011, as well as a number of associations for complex diseases unpublished at that time, variants in the human leukocyte antigen (HLA) region, and a scaffold of approximately 5,000 SNPs placed on the array for identity by descent testing. The results of an analysis of rare (minor-allele frequency <5%) coding sequence (“exome”) variants on this array with CAD were recently reported (5).

We identified 6 new loci associated at genome-wide significance with CAD, annotated these, and undertook a detailed examination of the extent of pleiotropy of these loci as well the previously known CAD loci.

## Methods

The study consisted of discovery and replication phases and has been described in more detail elsewhere (5). Briefly, the discovery cohort included 42,335 cases and 78,240 control subjects from 20 individual studies (Online Table 1); the replication cohort, which was separately assembled and ascertained to have no sample overlap with the discovery cohorts, included 30,533 cases and 42,530 control subjects from 8 studies (Online Table 2). With the exception of participants from 2 studies in the replication cohort who were of South Asian ancestry, all participants were of European ancestry (Online Table 2).

Samples were genotyped on the Illumina HumanExome BeadChip versions 1.0 or 1.1, or the Illumina OmniExome (which includes markers from the HumanExome BeadChip) arrays followed by quality control procedures as previously described (5).

### Statistical analysis

In discovery samples that passed quality control procedures, we performed individual tests for association of the selected variants with CAD in each study separately, using logistic regression analysis with principal components of ancestry as covariates (5). We combined evidence across individual studies using an inverse-variance weighted fixed-effects meta-analysis. Heterogeneity was assessed by Cochran’s Q statistic (6). In the discovery phase, we defined suggestive novel association as a meta-analysis p value ≤1 × 10^−6^.

For variants with suggestive association, we performed association analysis in the replication studies (Online Appendix). We defined significant novel associations as those nominally significant (p < 0.05) in the replication study and with an overall (discovery and replication combined) p value <5 × 10^−8^.

### Bioinformatics analysis

To identify any association between the novel loci and gene expression traits, we performed a systematic search of cis-expression quantitative trait loci (eQTL) (described in the Online Appendix). To identify candidate causal SNPs at the new loci, we annotated each of the lead variants as well as SNPs in high linkage disequilibrium (LD) (r^2^ > 0.8) on the basis of position, overlap with regulatory elements, and in silico SNP prioritization tools (Online Appendix).

For both the novel loci and all previously reported CAD loci 1, 2, we tested the association of the lead CAD-associated variant (or, if unavailable, a proxy) with traditional cardiovascular risk factors using publicly available GWAS meta-analyses datasets for systolic, diastolic, and pulse pressures 7, 8; low-density lipoprotein (LDL) cholesterol level; high-density lipoprotein (HDL) cholesterol level; triglycerides level 9, 10; type 2 diabetes mellitus (11); body mass index (BMI) (12); and smoking quantity (13). The maximum size of these datasets ranged from 41,150 to 339,224 individuals. For variants available on the exome array with a known genome-wide association with a risk factor, we also compared the magnitude of the reported association with the risk factor to the observed association with CAD in our analysis.

To identify any associations with other diseases or traits, we searched version 2 of the GRASP (Genome-Wide Repository of Associations between SNPs and Phenotypes) database (14) and the National Human Genome Research Institute-European Bioinformatics Institute GWAS catalog (15), plus we collected all associations below 1 × 10^−4^. For all associations, we identified the lead variant for that trait or disease and calculated pairwise LD with the lead CAD-associated variant using the SNAP web server (16).

## Results

In the discovery cohort, 28 variants not located in a known CAD locus (defined as ±300 kb from the published lead SNP) showed association with CAD at a p value <1 × 10^−6^ (Online Table 3). No marked heterogeneity was observed, justifying the use of a fixed-effects model. We then tested these 28 variants for replication, and 6 variants showed both a nominally significant (p < 0.05) association in the replication cohort and a combined discovery and replication meta-analyses p value exceeding the threshold for genome-wide significance (p < 5 × 10^−8^) (Table 1). As typical for GWAS findings, the risk alleles were common (allele frequencies ranging from 15% to 86%), and the risk increase per allele was modest (ranging from 4% to 9%) (Table 1).

### Annotation of novel loci

Forest and regional association plots for the 6 novel loci are shown in Online Figures 1 and 2, respectively. Interrogation of the 1000 Genomes Project phase 1 EUR data using Haploreg (BROAD Institute, Massachusetts Institute of Technology and Harvard, Boston, Massachusetts) (17) showed that the number of SNPs in high LD (r^2^ > 0.8) with the lead variant varied between 1 (*LRP1* locus and *CETP* locus) and 111 (*KCNJ13-GIGYF2* locus) (Online Table 4). Apart from the lead variant at the *KCNJ13-GIGYF2* locus, which is a nonsynonymous SNP, none of the other loci had a variant affecting protein sequence in high LD with the lead variant.

Notable cis-eQTL findings for the new loci are shown in Online Table 5 and functional annotation of the lead variant and variants in high LD appear in Online Figure 3. The main findings from these analyses are discussed here locus by locus.

#### 16q13

The lead variant, rs1800775, also known as −629C>A, is in the promoter of the cholesteryl ester transfer protein (*CETP)* gene, which mediates the transfer of cholesteryl esters from HDL cholesterol to other lipoproteins and was placed on the array because of its association with plasma HDL cholesterol level 9, 10. The risk (C) allele is associated with lower HDL cholesterol and modest increases in plasma LDL cholesterol and triglycerides levels 9, 10. Previous studies have shown that rs1800775 is itself functional in that the C allele disrupts binding of the Sp1 transcription factor resulting in increased promoter activity (18). This is in agreement with our annotation, which predicts this to be more likely to be a functional SNP than the only other SNP in high LD, rs3816117 (Online Figure 3). Consistent with this, we also found associations between rs1800775 and *CETP* expression (r^2^ of 0.77) with the best eSNP (i.e., the lead SNP for the eQTL) in monocytes and liver (Online Table 5), and previous studies have shown that the variant is also associated with plasma CETP level 19, 20.

#### 12q24

The lead variant, rs11057830, and all 8 variants in high LD are located in a region of approximately 10 kb in intron 1 of *SCARB1,* which encodes SR-B1, a receptor for HDL cholesterol. Other variants at this locus have been associated with HDL cholesterol level 9, 10. However, these HDL cholesterol variants are not in high LD with the CAD-associated variants identified here, which only have a modest association with plasma HDL cholesterol level (Online Table 6), but a stronger association with plasma LDL cholesterol and triglycerides levels (Table 2). rs11957830 was included on the array because of an association of the A allele (CAD risk-associated allele) with higher levels of vitamin E (Table 3) (21). Variants in high LD with the CAD risk allele at rs11057830 have also been associated with increased lipoprotein-associated phospholipase A_2_ (Lp-PLA_2_) activity (22). Analysis of eQTL identified an association between rs11057841 (r^2^ = 0.92 with the lead variant), and expression of *SCARB1* in the intestine (Online Table 5). Functional annotation of the locus did not identify a strong candidate causal SNP, but rs10846744 (r^2^ = 0.94 with the lead variant) overlaps a deoxyribonuclease I hypersensitivity peak in a region bound by several transcription factors (Online Figure 3).

#### 12q13

The lead variant, rs11172113, is in intron 1 of *LRP1* (LDL receptor–related protein-1) and only has 1 other adjacent SNP in high LD (Central Illustration, Online Table 4). The risk (C) allele of the lead variant has previously been associated with reduced risk of migraine (23), and there is an association of the alternate (T) allele with reduced lung function (24). There are also associations at this locus for abdominal aortic aneurysm (25) and triglyceride levels (10); however, these variants are in modest or low LD to the CAD-associated SNP (r^2^ of 0.54 and 0.07, respectively). The lead variant overlaps a region containing peaks in deoxyribonuclease I hypersensitivity in several cells and tissues, including aortic smooth muscle cells, within a predicted enhancer element (Online Figure 3). We found associations between the CAD risk allele at rs11172113 and reduced expression of *LRP1* in atherosclerotic and nonatherosclerotic arterial wall, as well as eQTLs in omental and subcutaneous adipose tissue (Online Table 5).

#### 11p15

The lead variant, rs11042937, at this locus lies in an intergenic region between *MRVI1* (murine retrovirus integration site 1 homolog) encoding inositol-trisphosphate receptor-associated cyclic guanosine monophosphate kinase substrate, a mediator of smooth muscle tone and *CTR9* that encodes a component of the PAF1 complex with some SNPs in high LD located within intron 1 of *MRVI1* (Online Figure 3). The lead variant was included on the array because of a suggestive association with bipolar disorder and schizophrenia (26). There was no association of the locus with any cardiovascular risk factors, and we did not identify any eQTLs. Evidence for a regulatory function for either the lead variant or any of the SNPs in high LD was also weak (Online Figure 3).

#### 6p21

The lead variant, rs3130683, lies in the HLA complex in intron 1 of *C2*, which encodes the complement C2 protein. There are just 14 SNPs in high LD with the lead variant (Online Table 4), but the CAD signal spans a region of approximately 300 kb including more than 20 genes (Online Figure 3). Apart from a single synonymous variant in *HSPA1A* (heat shock 70kDa protein 1A), the other high LD variants are noncoding with several of the variants showing evidence for regulatory functionality (Online Figure 3). Although there is a large number of eQTLs in the HLA region, most of these are variants with modest (r^2^ < 0.5) LD with the CAD-associated variants, and the only eQTL of note was with *CYP21A2* (cytochrome P450, family 21, subfamily A, polypeptide 2) expression in whole blood (Online Table 5). rs3869109, another variant at the HLA locus approximately 700 kb away from the new lead variant, has been reported to be associated with CAD (27). In our discovery cohort, rs3869109 has a p value of association with CAD of 0.23.

#### 2q37

The lead variant, rs1801251, was included on the array for identity by descent testing; rs1801251 causes a threonine to isoleucine amino acid change at position 95 in KCNJ13, an inwardly rectifying potassium channel protein. However, this is not predicted to be functionally important. There is extended linkage at this locus, with more than 100 SNPs in high LD and the lead variant in a region of ∼170 kb also spanning *GIGYF2* (GRB10 interacting GYF protein 2) (Online Figure 3). *KCJN13* is located entirely within *GIGYF2* and transcribed in the opposite direction. A number of the associated variants are in annotated regulatory regions, with the top scoring candidate by in silico prediction, rs11555646, lying in the 5′-UTR of GIGYF2 close to the initiating methionine (Online Figure 3). There was no association of the locus with any of the cardiovascular risk factors, but we found eQTLs for the lead variant or a variant in high LD for both *GIGFY2* and *KCNJ13* (Online Table 5).

### CAD loci and pleiotropy

We undertook an updated analysis of the association of all 62 CAD loci (56 published and 6 novel in this report) with traditional cardiovascular risk factors (blood pressure traits, lipid traits, BMI, type 2 diabetes, and smoking). The full results are shown in Online Table 6, and the significant associations are summarized in Table 2. Of the 62 CAD loci, 24 (38.7%) showed a statistical association at a Bonferroni corrected p value <8.32 × 10^−5^ with a traditional cardiovascular risk factor with some loci showing multiple associations (Central Illustration). The largest number of associations were with lipid traits (14 with LDL cholesterol, 9 with HDL cholesterol, and 7 with triglycerides), followed by blood pressure traits (5 with diastolic blood pressure, 4 with systolic blood pressure, and 1 with pulse pressure), BMI (5 associations), and type 2 diabetes (1 association). Most associations were in the direction consistent from the epidemiological association of these risk factors with CAD, although a few displayed effects in the opposite direction (the risk variants at 2q33 and 12q24 are associated with reduced plasma LDL cholesterol, and those at 10q24, 12q24, and 19q13 are associated with lower BMI).

To inform the interpretation of these data, we conducted a complementary analysis for variants available on the array with a known genome-wide association with a risk factor; also, we compared the magnitude of the reported association with the risk factor to the observed association with CAD in our data. Except for LDL cholesterol and BMI, the correlations between the 2 effects were either weak or insignificant (Online Figure 4). In a separate analysis conducted in the 150,000 participants in UK Biobank with currently released genotype data, we confirmed that none of the CAD-associated variants showed a sex difference in allele frequency (data not shown).

We next analyzed the association of the 62 CAD loci with other diseases and traits. When restricted to variants with a high LD (r^2^ > 0.8) with the lead CAD variant, 29 of 62 (47%) loci showed an association with another disease/trait at a p value <1 × 10^−4^. Several loci showed multiple associations (Table 3). Although in most cases, the CAD-associated risk allele was also associated with an increased risk (or level) of the other disease or trait, this was not always the case. Furthermore, in some loci with multiple associations, the direction of association varied between diseases (Table 3).

## Discussion

This large-scale meta-analysis of common variants, including many with prior evidence for association with another complex trait, resulted in the identification of 6 new CAD loci at genome-wide significance. We also showed that almost one-half of the CAD loci that have been identified to date demonstrate pleiotropy, an association with another disease or trait. The findings added to our understanding of the genetic basis of CAD and might provide clues to the mechanisms by which such loci affect CAD risk.

Our findings of a genome-wide association with CAD of a functional variant in the promoter of the *CETP* gene that is also associated with its expression and plasma activity 18, 19, 20 have added to previous evidence linking genetically determined increased activity of this gene with higher risk of CAD (20). There has been a longstanding interest in CETP inhibition as a therapeutic target, primarily because of the effect on plasma HDL cholesterol level. However, several CETP inhibitors have recently failed to improve cardiovascular outcomes in large randomized clinical trials 28, 29, 30 and, in 1 case, caused harm (28), despite markedly increasing plasma HDL cholesterol. Furthermore, Mendelian randomization studies have questioned the causal role of lower plasma HDL cholesterol in increasing CAD risk 31, 32. Although previous studies have shown that the CETP genetic variant we report here affects CETP activity, the precise mechanism(s) by which this variant modifies CAD risk remains uncertain.

A notable finding was the association with CAD of common variants located in the *SCARB1* gene. Association of variants at the *SCARB1* locus with CAD was also reported by the CARDIoGRAMplusC4D consortium, but this did not reach genome-wide significance (1). The gene encodes the canonical receptor, SR-BI, responsible for HDL cholesteryl ester uptake in hepatocytes and steroidogenic cells (33). Genetic modulation of SR-BI levels in mice is associated with marked changes in plasma HDL cholesterol (34). Consistent with this, a rare loss of function variant in which leucine replaces proline 376 (P376L) in *SCARB1* was recently identified through sequencing of individuals with high plasma HDL cholesterol (35). Interestingly, despite having higher plasma HDL, 346L carriers had an increased risk of CAD, suggesting that the association of variation at this locus on CAD is not driven primarily through plasma HDL (35). Indeed, there is only a nominal association of the lead CAD variant at this locus (rs11057830) with plasma HDL cholesterol (Online Table 6). The variant is also modestly associated with plasma LDL cholesterol and serum triglycerides (Table 2). All 3 of these lipid associations are directionally consistent with epidemiological evidence linking them to CAD risk and could, in combination, explain the association of the locus with CAD. However, the lead variant is more strongly associated with Lp-PLA_2_ activity and mass (Table 3), which could provide an alternative explanation for its association with CAD. Irrespective of the mechanism, our findings, when combined with those of Zanoni et al. (35), suggest that modulating SR-B1 may be therapeutically beneficial.

After adjusting for multiple testing, we found that slightly more than one-third of the CAD loci showed an association with traditional cardiovascular risk factors. Although the vast majority of associations were in the direction consistent with the epidemiological association of these risk factors with CAD, as noted in the previous text with respect to loci affecting the HDL cholesterol level, this should not be interpreted as implying that these loci affect CAD risk through an effect on the specific risk factor. Indeed, for variants available on the array with a known genome-wide association with these risk factors, we found a poor correlation between the magnitudes of their effect of the risk factor and their association with CAD in our dataset except for LDL cholesterol (Online Figure 4). Nonetheless, formal causal inference analyses, using Mendelian randomization, have implicated LDL cholesterol, triglyceride-rich lipoproteins, blood pressure, type 2 diabetes, and BMI as causally involved in CAD (36).

Almost one-half of the CAD loci showed a strong or suggestive association with other diseases or traits with, in many cases, the identical variant being the lead variant reported for the association with these other conditions (Table 3). Some of the associations with other traits—for example, coronary calcification (3q22, 6p24, 9p21, 13q34, and 15q25) or carotid intima-media thickness (4q31 and 19q13)—are not surprising, as these traits are known to be correlated with CAD. Others, such as risk of stroke (7p21 and 9p21), might reflect a shared etiology. However, the mechanism(s) behind most of the observed pleiotropy is not clear, although the findings could provide clues as to how the locus may affect CAD risk. As an example, 5 loci (12q24, 1p13, 6q25, 11q23, and 19q13) show strong associations with plasma activity and/or mass of Lp-PLA_2_. Lp-PLA_2_ is expressed in atherosclerotic plaques where studies have suggested a role in the production of proinflammatory and pro-apoptotic mediators, primarily through interaction with oxidized LDL 37, 38. A meta-analysis of prospective studies showed an independent and continuous relationship of plasma Lp-PLA_2_ with CAD risk (39). However, it should be noted that Mendelian randomization analyses have not supported a causal role of secreted Lp-PLA_2_ in coronary heart disease (40), and phase III trials of darapladib, an Lp-PLA_2_ inhibitor, have shown no benefit in patients with stable coronary heart disease (41) or acute coronary syndromes (42) when added to conventional treatments including statins.

Chronic inflammation plays a key role in both the pathogenesis of CAD and of inflammatory bowel disease. It is therefore interesting to note the association of the same locus at 15q22 with CAD as well as Crohn’s disease and ulcerative colitis (Table 3). Association of this locus with CAD at genome-wide significance was recently reported by the CARDIoGRAMplusC4D consortium (2) with the lead SNP (rs56062135) showing strong linkage disequilibrium (r^2^ = 0.9) with the lead SNP (rs17293632) associated with inflammatory bowel disease. Both rs56062135 and rs17293632 lie in a region of ∼30 kb within the initial introns of the SMAD family member 3 gene (*SMAD3*), a signal transducer in the transforming growth factor–beta pathway. Indeed, rs17293632 was included on the exome array because of its known association with Crohn’s disease and showed a significant association with CAD in our combined dataset (p = 1.78 × 10^−8^). Farh et al. (43) interrogated ChIP-seq data from ENCODE and found allele-specific binding of the AP-1 transcription factor to the major (C) allele in heterozygous cell lines and suggested that the T allele of rs17293632 increases risk of Crohn’s disease by disrupting AP-1 regulation of *SMAD3* expression. Interestingly, the direction of effect on CAD risk observed for this variant was in the opposite direction to that for inflammatory disorders, with the C allele being the risk allele. Recent analysis of this variant in arterial smooth muscle cells confirmed that the CAD risk allele preserves AP-1 transcription factor binding and increases expression of *SMAD3* (44). Further investigation of the discordant effects of SMAD3 may shed light on the mechanisms of both diseases.

### Study limitations

First, in our discovery study, we were only able to interrogate common variants associated with other diseases and traits that were known at the time of the creation of the exome array in late 2011 and, thus, included on the array. Conversely, our interrogation for pleiotropic associations of the new and known CAD has used the latest data available in the GWAS catalogs and other sources. Second, the common variants tested in our study conferred statistically robust yet quantitatively modest effects on both CAD and potentially related traits. Thus, we may have missed associations with other traits. However, if such traits were considered as intermediary steps in the etiology of CAD, exploration of our large GWAS sample sets and respective GWAS catalogs should have detected relevant associations. Third, our discovery analysis is largely on the basis of subjects with Western-European ancestry, and any association with CAD of the new loci in other populations needs further evaluation. Finally, although we used relatively stringent criteria (minimal r^2^ > 0.8 between the CAD SNP and the lead variant associated with the other disease/trait), the limited content of the exome array and the information available in the GWAS catalogs meant that we could not examine the extent of overlap in the loci in detail.

## Conclusions

Through an analysis of selected variants associated with other disease traits, we reported the discovery of 6 further loci associated with CAD. Furthermore, in the most comprehensive analysis to date, we showed that several of the new and previously established loci demonstrated substantial pleiotropy, which may help our understanding of the mechanisms by which these loci affect CAD risk.

Perspectives **COMPETENCY IN MEDICAL KNOWLEDGE:** Novel genetic loci influence risk of coronary artery disease, but only one-third are associated with conventional cardiovascular risk factors, whereas at least one-half of the loci are associated with other diseases or traits (pleiotropy).

**TRANSLATIONAL OUTLOOK:** Future studies should investigate the mechanisms that relate the observed pleiotropy to the pathogenesis of atherosclerosis and ischemic events.

## Figures and Tables

**Central Illustration:**
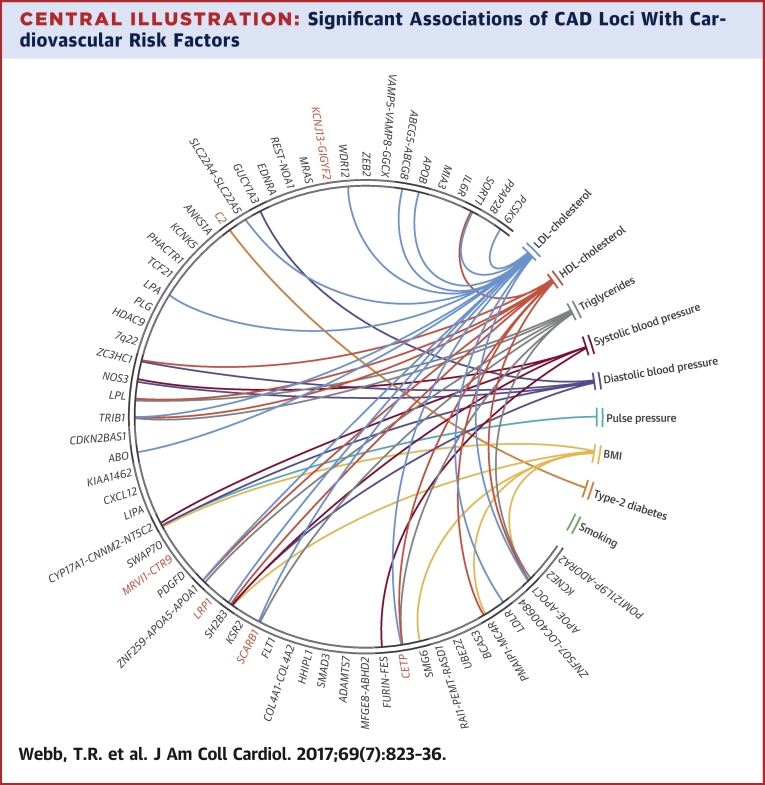
Significant Associations of CAD Loci With Cardiovascular Risk Factors This chord diagram depicts associations that passed Bonferroni correction (Table 2). Connections indicate that single nucleotide polymorphisms at respective loci associate with both coronary artery disease (CAD) and the respective risk factor; they do not imply that the risk factor causally explains the association with CAD. **Red** indicates new CAD loci. BMI = body mass index; HDL = high-density lipoprotein; LDL = low-density lipoprotein.

**Table 1:** Novel Loci Significantly Associated With CAD

Lead Variant	Locus	Locus Name	A1/2 (Freq)	Discovery			Replication			Meta-Analysis		Known Associations With Lead Variant
				Cases/Control Subjects	OR (95% CI)	p Value	Cases/Control Subjects	OR (95% CI)	p Value	OR (CI 95%)	p Value	
rs1801251	2q37	*KCNJ13-GIGYF2*	A/G (0.35)	42,332/78,229	1.06 (1.04–1.08)	1.46 × 10^−8^	30,528/42,521	1.03 (1.01–1.06)	0.007	1.05 (1.03–1.06)	1.48 × 10^−9^	—
rs3130683	6p21	*C2*	T/C (0.86)	39,494/72,267	1.09 (1.06–1.13)	7.87 × 10^−8^	30,450/42,485	1.09 (1.05–1.14)	2.97 × 10^**−**5^	1.09 (1.07–1.12)	1.04 × 10^−11^	—
rs11042937	11p15	*MRVI1-CTR9*	T/G (0.49)	42,335/78,234	1.05 (1.03–1.07)	3.21 × 10^−8^	30,533/42,527	1.03 (1.00–1.05)	0.019	1.04 (1.03–1.06)	1.18 × 10^−8^	Bipolar disorder and schizophrenia
rs11172113	12q13	*LRP1*	C/T (0.41)	42,335/78,234	1.06 (1.04–1.08)	1.78 × 10^−8^	28,503/36,433	1.06 (1.03–1.08)	1.16 × 10^−6^	1.06 (1.04–1.07)	9.25 × 10^−14^	Migraine, pulmonary function
rs11057830	12q24	*SCARB1*	A/G (0.15)	42,331/78,237	1.09 (1.06–1.11)	3.69 × 10^−10^	20,395/30,592	1.07 (1.03–1.11)	0.0003	1.08 (1.06–1.10)	4.61 × 10^−13^	Vitamin E level
rs1800775	16q13	*CETP*	C/A (0.51)	38,810/62,756	1.06 (1.04–1.08)	2.21 × 10^−8^	22,445/32,148	1.03 (1.00–1.05)	0.032	1.04 (1.03–1.06)	9.83 × 10^−9^	HDL cholesterol

A1/2 = allele 1/allele 2; CAD = coronary artery disease; CI = confidence interval; freq = frequency of allele 1; HDL = high-density lipoprotein; OR = odds ratio for disease for carriers of allele 1.

**Table 2:** Significant Associations of CAD Variants With Selected CV Risk Factors∗

Locus	Locus Name	Lead Variant	Trait	Effect	p Value
New Loci					
6p21	*C2*	rs3130683	T2D	1.12†	2.7 × 10^−5^
	*SCARB1*	rs11057830	LDL	0.006	2.6 × 10^−5^
			TG	0.022	8.3 × 10^−5^
16q13	*CETP*	rs1800775	LDL	0.041	8.5 × 10^−24^
			HDL	−0.202	3.3 × 10^−644^
			TG	0.04	1.3 × 10^−26^

Known Loci					
1p32	*PCSK9*	rs11206510	LDL	0.083	2.4 × 10^−53^
1p13	*SORT1*	rs602633	LDL	0.159	1.5 × 10^−261^
			HDL	−0.033	3.5 × 10^−14^
2p24	*APOB*	rs515135	LDL	0.139	1.1 × 10^−178^
2p21	*ABCG5-ABCG8*	rs6544713	LDL	0.081	4.84 × 10^−83^
4q32	*GUCY1A3*	rs7692387	DBP	0.326	3.4 × 10^−5^
5q31	*SLC22A4-SLC22A5*	rs273909	LDL	0.022	2.3 × 10^−5^
2q33	*WDR12*	rs6725887	LDL	−0.026	1.3 × 10^−5^
6q25	*LPA*	rs3798220	LDL	0.158	6.1 × 10^−11^
		rs2048327	LDL	0.019	1.3 × 10^−6^
7q32	*ZC3HC1*	rs11556924	DBP	NA	1.8 × 10^−5^
			HDL	−0.018	1.3 × 10^−5^
7q36	*NOS3*	rs3918226	SBP	0.96	1.1 × 10^−6^
			DBP	0.81	2.2 × 10^−9^
8p21	*LPL*	rs264	HDL	−0.098	8 × 10^−77^
			TG	0.093	2.4 × 10^−84^
8q24	*TRIB1*	rs2954029	LDL	0.056	2.1 × 10^−50^
			HDL	−0.04	2.7 × 10^−29^
			TG	0.076	1 × 10^−107^
9q34	*ABO*	rs579459	LDL	0.067	2.4 × 10^−44^
10q24	*CYP17A1-CNNM2-NT5C2*	rs12413409	SBP	1.034	2 × 10^−9^
			DBP	0.483	3.4 × 10^−5^
			PP	0.56	5.7 × 10^−8^
			BMI	−0.03	2.2 × 10^−8^
11q23	*ZNF259-APOA5-APOA1*	rs964184	LDL	0.086	2 × 10^−26^
			HDL	−0.107	6.1 × 10^−48^
			TG	0.234	6.6 × 10^−244^
12q24	*SH2B3*	rs3184504	LDL	−0.027	4.2 × 10^−12^
			HDL	−0.026	4.1 × 10^−12^
			SBP	0.598	2 × 10^−9^
			DBP	0.483	8.8 × 10^−6^
			BMI	−0.131	9.4 × 10^−6^
15q26	*FURIN-FES*	rs17514846	SBP	0.509	1.2 × 10^−5^
17p13	*SMG6*	rs2281727	BMI	0.015	3.64 × 10^−6^
18q21	*PMAIP1-MC4R*	rs663129	HDL	−0.026	5.5 × 10^−9^
			BMI	0.056	8.8 × 10^−53^
19p13	*LDLR*	rs1122608	LDL	0.074	8.5 × 10^−57^
19q13	*APOE-APOC1*	rs2075650	LDL	0.177	1.7 × 10^−214^
			HDL	−0.055	9.7 × 10^−26^
			TG	0.044	2.3 × 10^−21^
			BMI	−0.026	1.3 × 10^−8^
		rs445925	LDL	0.363	6.6 × 10^−397^
			HDL	−0.051	1.9 × 10^−10^
			TG	0.101	3.6 × 10^−39^

BMI = body mass index; CAD = coronary artery disease; CV = cardiovascular; DBP = diastolic blood pressure; HDL = high-density lipoprotein; LDL = low-density lipoprotein cholesterol level; T2D = type 2 diabetes mellitus; TG = triglycerides.

∗ Effects are either absolute beta estimates of the association of the CAD risk allele on the trait (with a positive association indicating a higher value of the trait per copy of the risk allele) or log odds ratio for diabetes mellitus, †per copy of the risk allele. This table only includes associations that passed Bonferroni correction.

**Table 3:** Association of CAD Loci With Other Diseases or Traits

Locus	Locus Name	Disease or Trait	Disease or Trait Lead SNP	p Value	Direction	r2 (CAD Lead and Disease or Trait Lead)
New Loci						
6p21	*C2*	Systemic lupus erythematosus	rs3130342	9.3 × 10^−7^	+	0.87
		Primary biliary cirrhosis	rs3134954	1 × 10^−5^	+	0.86
		Multiple sclerosis	rs3134954	3.2 × 10^−9^	−	0.86
11p15	*MRVI1-CTR9*	Bipolar disorder and schizophrenia	rs2018368	1 × 10^−6^	+	0.96
12q13	*LRP1*	Migraine	rs11172113	4.3 × 10^−9^	−	Same SNP
		Lung function (FEV1/FVC)	rs11172113	1.2 × 10^−8^	+	Same SNP
		Cervical artery dissection	rs11172113	3 × 10^−7^	−	Same SNP
12q24	*SCARB1*	Lp-PLA_2_ activity	rs11057841	6.1 × 10^−14^	+	0.92
		Circulating vitamin E levels	rs11057830	8.2 × 10^−9^	+	Same SNP
		Lp-PLA_2_ mass	rs10846744	6.8 × 10^−6^	+	0.94

Known Loci						
1p32	*SORT1*	Lp-PLA_2_ activity	rs7528419	1.3 × 10^−17^	+	0.9
		Metabolic syndrome domains (Atherogenic dyslipidemia–PC1)	rs12740374	8 × 10^−16^	+	Same SNP
		Lp-PLA_2_ mass	rs7528419	7.1 × 10^−5^	+	0.9
1q21	*IL6R*	C-reactive protein	rs4845625	4.2 × 10^−7^	+	Same SNP
2p21	*ABCG5-ABCG8*	Serum phytosterol	rs4245791	2.2 × 10^−70^	+	1
2p11	*VAMP5-VAMP8-GGCX*	Prostate cancer	rs10187424	2.7 × 10^−15^	−	0.87
2q33	*WDR12*	Cerebral white matter hyperintensities burden	rs6705330	5.7 × 10^−5^	+	1
3q22	*MRAS*	Coronary artery calcification	rs2306374	2.7 × 10^−5^	+	1
4q12	*REST-NOA1*	Height	rs17081935	6.7 × 10^−17^	+	0.95
4q31	*EDNRA*	Carotid intima media thickness	rs1878406	7 × 10^−12^	+	Same SNP
		Intracranial aneurysm	rs6842241	2.4 × 10^−9^	+	0.94
6p24	*PHACTR1*	Coronary artery calcification	rs9349379	4 × 10^−22^	+	Same SNP
		Cervical artery dissection	rs9349379	1 × 10^−11^	−	Same SNP
		Migraine	rs9349379	5 × 10^−8^	−	Same SNP
		Pulse wave velocity	rs7750679	5.4 × 10^−5^	+	1
6q25	*LPA*	Lipoprotein (a)	rs3798220	1.6 × 10^−49^	+	Same SNP
		Colorectal cancer	rs7758229	5.6 × 10^−9^	−	0.85
7p21	*HDAC9*	Stroke (large vessel stroke)	rs11984041	1.9 × 10^−11^	+	1
7q22	7q22	Endometriosis	rs10953541	3.2 × 10^−5^	+	Same SNP
8q24	*TRIB1*	Metabolic syndrome domains (Atherogenic dyslipidemia–PC1)	rs2954021	1.2 × 10^−11^	+	Same SNP
		Adiponectin levels	rs2954021	1.8 × 10^−5^	+	Same SNP
		Serum creatinine	rs2954021	2.3 × 10^−5^	+	Same SNP
9p21	*CDKN2BAS1*	Coronary artery calcification	rs1333049	3.3 × 10^−24^	+	0.97
		Abdominal aortic aneurysm	rs2383207	1.9 × 10^−8^	+	0.91
		Ankle brachial index	rs10757269	2.7 × 10^−9^	+	0.9
		Stroke (large vessel stroke)	rs2383207	2.4 × 10^−6^	+	0.91
		Intracranial aneurysm	rs10733376	4 × 10^−12^	+	0.94
9q34	*ABO*	Alkaline phosphatase in plasma	rs579459	3 × 10^−123^	+	Same SNP
		Activated partial thromboplastin time	rs579459	1.7 × 10^−74^	+	Same SNP
		Soluble P-selectin	rs579459	1.9 × 10^−41^	+	Same SNP
		Soluble E-selectin	rs579459	1.3 × 10^−29^	+	Same SNP
		Plasma carcinoembryonic levels	rs579459	3 × 10^−21^	+	Same SNP
		Red blood cell count	rs579459	9.3 × 10^−18^	+	Same SNP
		Hemoglobin	rs579459	1.4 × 10^−15^	+	Same SNP
		Hematocrit	rs579459	7.6 × 10^−14^	+	Same SNP
		Interleukin-6 levels	rs579459	3.6 × 10^−13^	+	Same SNP
		Circulating galectin-3 levels	rs579459	1.9 × 10^−10^	+	Same SNP
		Factor XIII antigen	rs579459	2.3 × 10^−8^	+	Same SNP
		von Willebrand factor	rs651007	1 × 10^−161^	+	1
		Venous thromboembolism	rs495828	2 × 10^−17^	+	1
		Serum alkaline phosphatase levels	rs651007	1 × 10^−56^	+	1
		Ferritin levels	rs651007	1 × 10^−8^	+	1
		Soluble ICAM-1	rs507666	3 × 10^−91^	+	0.83
10q24	*CYP17A1-CNNM2-NT5C2*	Intracranial aneurysm	rs12413409	1.2 × 10^−9^	+	Same SNP
		Schizophrenia	rs11191580	1.7 × 10^−9^	+	1
		Autism spectrum disorder	rs11191454	1.4 × 10^−8^	+	1
		Parkinson's disease	rs17115100	7.4 × 10^−8^	+	0.9
11q23	*ZNF259-APOA5-APOA1*	Metabolic syndrome domains (Atherogenic dyslipidemia–PC2)	rs964184	1.8 × 10^−12^	+	Same SNP
		Vitamin E levels	rs964184	7.8 × 10^−12^	+	Same SNP
		Lp-PLA_2_ activity	rs964184	8.4 × 10^−11^	+	Same SNP
		Metabolic syndrome domains (Atherogenic dyslipidemia–PC1)	rs964184	1.2 × 10^−10^	+	Same SNP
12q24	*SH2B3*	Selective immunoglobulin A deficiency	rs3184504	5.6 × 10^−31^	+	Same SNP
		Type 1 diabetes	rs3184504	2.8 × 10^−27^	+	Same SNP
		Celiac disease	rs3184504	5.4 × 10^−21^	+	Same SNP
		Hemoglobin	rs3184504	4.3 × 10^−19^	+	Same SNP
		Celiac disease	rs3184504	5.4 × 10^−21^	+	Same SNP
		Blood eosinophil count	rs3184504	6.5 × 10^−19^	+	Same SNP
		Generalized vitiligo	rs3184504	2.5 × 10^−17^	+	0.97
		Soluble ICAM-1	rs3184504	2.9 × 10^−17^	+	Same SNP
		Hematocrit	rs3184504	7.9 × 10^−16^	+	Same SNP
		Hypothyroidism	rs3184504	2.6 × 10^−12^	+	Same SNP
		Rheumatoid arthritis and celiac disease	rs3184504	1.4 × 10^−11^	+	Same SNP
		Primary sclerosing cholangitis	rs3184504	5.9 × 10^−11^	+	Same SNP
		Serum urate	rs3184504	2.6 × 10^−10^	+	Same SNP
		Juvenile idiopathic arthritis	rs3184504	2.6 × 10^−9^	+	0.9
		Lymphocyte count	rs3184504	1.1 × 10^−8^	+	Same SNP
		Plasma beta-2 microglobulin levels	rs3184504	3.1 × 10^−8^	+	Same SNP
		White blood cell count	rs3184504	6.3 × 10^−6^	+	Same SNP
		Rheumatoid arthritis	rs3184504	6 × 10^−6^	+	Same SNP
		Tetralogy of Fallot	rs11065987	4.6 × 10^−8^	+	0.91
13q34	*COL4A1-COL4A2*	Coronary artery calcification	rs3809346	8.6 × 10^−7^	+	0.97
15q22	*SMAD3*	Crohn's disease	rs17293632	2.7 × 10^−19^	−	0.9
		Inflammatory bowel disease	rs17293632	6 × 10^−16^	−	0.9
		Ulcerative colitis	rs17293632	9.5 × 10^−6^	−	0.9
		Self-reported allergy	rs17228058	1.2 × 10^−8^	−	0.9
15q25	*ADAMTS7*	Coronary artery calcification	rs3825807	6.5 × 10^−6^	+	Same SNP
17p13	*SMG6*	Aortic root size	rs10852932	2.3 × 10^−11^	+	0.96
17q21	*UBE2Z*	Height	rs318095	1.5 × 10^−16^	−	1
		Breast size (bra cup size in women)	rs12603969	3 × 10^−5^	−	1
18q21	*PMAIP1-MC4R*	Obesity	rs17782313	4.8 × 10^−15^	+	0.86
		Height	rs11152213	6.9 × 10^−13^	+	0.91
		Antipsychotic drug induced weight gain	rs12967878	3.6 × 10^−7^	+	0.9
19q13	*APOE-APOC1*	Common carotid artery intima-media thickness	rs445925	1.7 × 10^−8^	+	Same SNP
		Lp-PLA_2_ activity	rs445925	3.3 × 10^−10^	+	Same SNP
		Metabolic syndrome domains (Atherogenic dyslipidemia–PC1)	rs445925	1.3 × 10^−35^	+	Same SNP
		Alzheimer disease	rs2075650	1 × 10^−295^	+	Same SNP
		Longevity	rs2075650	3.4 × 10^−17^	−	Same SNP
		Lp-PLA_2_ activity	rs2075650	8.1 × 10^−15^	+	Same SNP
		Age-related macular degeneration	rs2075650	8.4 × 10^−8^	−	Same SNP
		C-reactive protein	rs2075650	4.2 × 10^−8^	+	Same SNP
		Cognitive decline	rs2075650	2 × 10^−8^	+	Same SNP

+ indicates increase in disease or trait with the CAD risk allele.

CAD = coronary artery disease; FEV1 = forced expiratory volume 1; FVC = forced vital capacity; ICAM-1 = intercellular adhesion molecule 1; Lp-PLA_2_ = lipoprotein-associated phospholipase A_2_; PC = principal component; SNP = single nucleotide polymorphism.
