# Supplementary material for: Systematic Evaluation of Pleiotropy Identifies 6 Further Loci Associated With Coronary Artery Disease
Source: J Am Coll Cardiol. 2017 Feb 21;69(7):823–36. doi: 10.1016/j.jacc.2016.11.056 (PMC5314135; doi:10.1016/j.jacc.2016.11.056)
Supplement: Online Data [file mmc1.docx]

**ONLINE APPENDIX**

**Acknowledgements**

TR Webb, and NJ Samani are funded by the British Heart Foundation, and NJ Samani is an NIHR Senior Investigator. N Stitziel is supported, in part, by a career development award from the NIH/NHLBI (K08HL114642) and by The Foundation for Barnes-Jewish Hospital. S Kathiresan is supported by a Research Scholar award from the Massachusetts General Hospital, the Donovan Family Foundation, grants from the NIH (R01HL107816 and R01HL127564), a grant from Fondation Leducq, and an investigator-initiated grant from Merck. P Deloukas’s work forms part of the research themes contributing to the translational research portfolio of Barts Cardiovascular Biomedical Research Unit, which is supported and funded by the National Institute for Health Research (NIHR). The analysis was funded, in part, by a Programme Grant from the BHF (RG/14/5/30893 to P Deloukas). GM Peloso is supported by the National Heart, Lung, and Blood Institute of the NIH (award number K01HL125751) PA Merlini was supported by a grant from the Italian Ministry of Health (RFPS-2007-3-644382). D Ardissino and N Marziliano were supported by Regione Emilia Romagna Area 1 Grants. M Farrall and H Watkins acknowledge the support of the Wellcome Trust core award (090532/Z/09/Z), the British Heart Foundation (BHF) Centre of Research Excellence. UM Schick is supported in part by a grant from the National Cancer Institute (R25CA094880). A Goel acknowledges EU FP7 & Wellcome Trust Institutional strategic support fund. NGD Masca was supported by the NIHR Leicester Cardiovascular Biomedical Research Unit (BRU), and this work forms part of the portfolio of research supported by the BRU. H-H Won was supported by a postdoctoral award from the American Heart Association (15POST23280019). MI McCarthy is a Wellcome Trust Senior Investigator (098381) and an NIHR Senior Investigator. J Danesh is a British Heart Foundation Professor, European Research Council Senior Investigator, and NIHR Senior Investigator. J Erdmann, TR Webb, NJ Samani, and H Schunkert are supported by the FP7 European Union project CVgenes@target (261123) and the Fondation Leducq (CADgenomics, 12CVD02). J Erdmann and H Schunkert are also supported by the German Federal Ministry of Education and Research e:Med program (e:AtheroSysMed and sysINFLAME), and Deutsche Forschungsgemeinschaft cluster of excellence “Inflammation at Interfaces” and SFB 1123. T Kessler received a DZHK Rotation Grant. GK Hovingh is a recipient of a VIDI grant (016.156.445) from the Netherlands Organisation for Scientific Research (NWO) and is supported by a grant from the CardioVascular Research Initiative (CVON2011-19; Genius) and the European Union (TransCard: FP7-603091-2).

The Exome Sequencing Project of the U.S. National Heart, Lung, and Blood Institute supported genotyping (RC2HL102925 to S Gabriel and D Altshuler). The study was also supported by the German Federal Ministry of Education and Research (BMBF) in the context of the e:Med program (e:AtheroSysMed) and the FP7 European Union project CVgenes@target (261123). Further grants were received by the Fondation Leducq (CADgenomics: Understanding CAD Genes, 12CVD02). This work has been supported by the “Programma di ricerca Regione-Università, Regione Emilia-Romagna, bando Ricerca Innovativa 2010-2012 to Dr. Diego Ardissino, Cardiovascular genetics: from bench to bedside - Genomic & transcriptomic of ischemic heart disease - CUP E35E09000880002”. The PopGen 2.0 network is supported by a grant from the German Ministry for Education and Research (01EY1103). Recruitment of the BHF-FHS Study was funded by the British Heart Foundation (BHF) with additional support from the Medical Research Council. Genotyping of the BHF-FHS controls was funded by the Wellcome Trust (through the Wellcome Trust Case Control Consortium, WTCCC) and the cases by the WTCCC, the National Institute for Health Research (NIHR) and the BHF. Data were obtained from Vanderbilt University Medical Center’s BioVU which is supported by institutional funding and by the Vanderbilt CTSA grant UL1 TR000445 from NCATS/NIH. This work was also in part supported by NIH grants U19 HL65962, and R01 HL092217. The Verona Heart Study is supported by the CariVerona Foundation. PROCARDIS was supported by the European Community Sixth Framework Program (LSHM-CT- 2007-037273), AstraZeneca, the British Heart Foundation, the Swedish Research Council, the Knut and Alice Wallenberg Foundation, the Swedish Heart-Lung Foundation, the Torsten and Ragnar Söderberg Foundation, the Strategic Cardiovascular Program of Karolinska Institutet and Stockholm County Council, the Foundation for Strategic Research and the Stockholm County Council (560283). EGCUT received financing from European Regional Development Fund, road-map grant no.3.2.0304.11-0312 and grant "Center of Excellence in Genomics" (EXCEGEN). EGCUT studies were covered also by targeted financing from Estonian Government (IUT24-6, IUT20-60) and CTG grant (SP1GVARENG) from Development Fund of the University of Tartu. GoDARTS acknowledges the support of the Health Informatics Centre, University of Dundee for managing and supplying the anonymized data and NHS Tayside, the original data owner. We are grateful to all the participants who enrolled in the GoDARTS study, to the general practitioners, to the Scottish School of Primary Care for their help in recruiting the participants, and to the whole team, which includes interviewers, computer and laboratory technicians, clerical workers, research scientists, volunteers, managers, receptionists, and nurses. The GoDARTS study is supported by the Wellcome Trust (Awards 072960, 084726 and 104970). The 1958 Birth Cohort sample collection was funded by the Medical Research Council grant G0000934 and the Wellcome Trust grant 068545/Z/02 and genotyping was funded by the Wellcome Trust. Jansson J-H was responsible for the identification of MI cases in the FIA3 study. The FIA3 study was supported in part by a grant from the Swedish Heart-Lund Foundation (grant no. 2020389 to Franks PW). Analysis was in part funded by BHF Programme Grant RG/14/5/30893 to P Deloukas. The KORA research platform (KORA, Cooperative Research in the Region of Augsburg) was initiated and financed by the Helmholtz Zentrum München - German Research Center for Environmental Health, which is funded by the German Federal Ministry of Education and Research and by the State of Bavaria. Furthermore, KORA research was supported within the Munich Center of Health Sciences (MC Health), Ludwig-Maximilians-Universität, as part of LMUinnovativ. We thank the Heinz Nixdorf Foundation (Germany), the Ministerium für Innovation, Wissenschaft und Forschung des Landes Nordrhein-Westfalen and the Faculty of Medicine University Duisburg-Essen for the generous support of the Heinz Nixdorf Recall Study. The BRAVE study genetic epidemiology working group is a collaboration between the Cardiovascular Epidemiology Unit, Department of Public Health and Primary Care, University of Cambridge, UK, the Centre for Control of Chronic Diseases, icddr,b, Dhaka, Bangladesh and the National Institute of Cardiovascular Diseases, Dhaka, Bangladesh. CHD case ascertainment and validation, genotyping, and clinical chemistry assays in EPIC-CVD were principally supported by grants awarded to the University of Cambridge from the EU Framework Programme 7 (HEALTH-F2-2012-279233), the UK Medical Research Council (G0800270) and British Heart Foundation (SP/09/002) , and the European Research Council (268834). We thank all EPIC participants and staff for their contribution to the study, the laboratory teams at the Medical Research Council Epidemiology Unit for sample management and Cambridge Genomic Services for genotyping, Sarah Spackman for data management, and the team at the EPIC-CVD Coordinating Centre for study coordination and administration. Field-work, genotyping, and standard clinical chemistry assays in PROMIS were principally supported by grants awarded to the University of Cambridge from the British Heart Foundation, UK Medical Research Council, Wellcome Trust, EU Framework 6–funded Bloodomics Integrated Project, Pfizer, Novartis, and Merck. The MORGAM Project received funding during the work from European Union FP 7 projects CHANCES (HEALTH-F3-2010-242244) and BiomarCaRE (278913). This has supported central coordination and part of the activities of the MORGAM Data Centre, at THL in Helsinki, Finland. MORGAM Participating Centres are funded by regional and national governments, research councils, charities, and other local sources. The Ottawa Heart Genomics Study was funded by Canadian Institutes of Health Research # MOP-2380941, #MOP82810, #MOP77682, Canada Foundation for Innovation #11966, Heart & Stroke Foundation of Canada T7268. The research leading to these results has received funding from the European Union's Seventh Framework Programme (FP7/2007-2013) under grant agreement n° HEALTH-F2-2009- 223004. The WHI program is funded by the National Heart, Lung, and Blood Institute, National Institutes of Health, U.S. Department of Health and Human Services through contracts HHSN268201100046C, HHSN268201100001C, HHSN268201100002C, HHSN268201100003C, HHSN268201100004C, and HHSN271201100004C. The authors thank the WHI investigators and staff for their dedication, and the study participants for making the program possible. A full listing of WHI investigators can be found at: http://www.whi.org/researchers/Documents%20%20Write%20a%20Paper/ WHI%20Investigator%20Short%20List.pdf. Exome-chip data and analysis were supported through the Women’s Health Initiative Sequencing Project (NHLBI RC2 HL- 102924), the Genetics and Epidemiology of Colorectal Cancer Consortium (NCI CA137088), the Genomics and Randomized Trials Network (NHGRI U01-HG005152), and an NCI training grant (R25CA094880). Malmö Diet and Cancer Study: Supported by the Swedish Research Council, the Swedish Heart and Lung Foundation, ERC-Stg- 282255, the Novo Nordic Foundation, the Swedish Diabetes Foundation, and the Påhlsson Foundation, and by equipment grants from the Knut and Alice Wallenberg Foundation, the Region Skåne, Skåne University Hospital, and the Linneus Foundation for the Lund University Diabetes Center. The work was funded as part of the DZHK and the eAtheroSysMed project BMBF 1ZX1313C. This work was also supported by grants from the European Union (CVgenes@target), the Leducq Foundation (CADgenomics), and the Bundesministerium für Bildung und Forschung (e:AtheroSysMed) to Jeanette Erdmann, Nilesh Samani, Hugh Watkins and/or Heribert Schunkert.

**Supplemental Methods**

**Statistical analysis**

In each replication study individually, we tested for association with CAD using a linear mixed model with fixed effects of genotype and principal components of ancestry and a kinship matrix as random effects. Results were combined within ancestry groups and then across all studies using inverse-variance weighted fixed effects meta-analyses.

**Bioinformatics analysis**

***Annotation of novel loci:*** To identify any association between novel loci and gene expression traits we performed a systematic search for expression quantitative trait loci (eQTLs) in the monocyte and macrophage expression study from the Cardiogenics consortium (1), the Stockholm-Tartu Atherosclerosis Reverse Network Engineering Task (STARNET) RNA-seq dataset (2) and over 100 studies included in the GRASP database (3). To identify candidate causal SNPs at the new loci we functionally annotated each of the lead variants as well all SNPs in high linkage disequilibrium (LD) SNPs (*r^2^* > 0.8) using Haploreg V3 (4). Non-synonymous variants were analyzed using SIFT (5) and Polyphen-2 (6). Conservation was assessed by GERP (7) and SiPhy (8). Overlap with regulatory elements including chromosome state segmentation, DNase hypersensitivity, and transcription factor binding as determined by the ENCODE (9) and Roadmap Epigenome projects (10), and predicted effects on transcription factor binding based on regulatory motifs from TRANSFAC (11) and JASPAR (12) were identified using Haploreg V3 (4) and the UCSC genome browser. Variants were then scored using three different bioinformatics tools that help prioritize causal disease variants. Combined Annotation Dependent Depletion (CADD) (13) incorporates a range pathogenicity prediction tools to provide a genome-wide score (C-score) for each test variant from its pre-calculated database of ~8.6 billion genetic variants. High scores indicate variants that are not stabilized by selection and are more likely to be disease-causing and low scores indicate evolutionary stable non-damaging variants. The top 10% of likely functional variants will have a C-score >10 and top 1% of variants will have a C-score >20. Genome-wide annotation of variants (GWAVA) (14) predicts the functional impact of noncoding variants based on a number of genomic and epigenomic annotations and provides scores between 0 and 1 with higher scores indicating variants that are more likely to be functional. RegulomeDB (15) annotates and scores variants based on a number of datasets, including ENCODE, and scores variants in seven categories. Scores of 1 and 2 identify variants that are likely to affect transcription factor binding, a score of 3 identifies variants that are less likely to affect binding, scores of 4, 5 and 6 relate to variants with minimal binding evidence and a score of 7 is for variants with no regulatory annotation.

**Supplemental Table 1: Sources of cases and controls in the discovery study**

| Study | Design | Case definition | Control definition | Cases | Controls | Reference | |
| --- | --- | --- | --- | --- | --- | --- | --- |
| ATVB | Case-control | MI in men or women ≤ 45 years of age | No history of thromboembolic disease | 1,428 | 1,069 | [^1^](#_ENREF_8)^6^ |  |
|  |  |  |  |  |  |  |  |
| BHF-FHS | Case-control | CAD cases were recruited from the British Heart Foundation Family Heart Study and supplemented by additional cases from WTCCC-CAD2 | Controls were selected from the UK 1958 Birth Cohort | 2,833 | 5,912 | ^17,18^ |  |
|  |  |  |  |  |  |  |  |
| BioVU | Case-control | Cases with MI or CAD were ascertained from the Vanderbilt University Medical Center Biorepository by searching the electronic medical record for ≥ 2 instances of ICD-9 codes 410.x – 414.x | Controls were individuals from the Vanderbilt University Biorepository who did not have any record of ICD-9 codes 410.x – 414.x | 4,587 | 16,556 | ^19^ |  |
|  |  |  |  |  |  |  |  |
| Duke | Case-control | MI or coronary stenosis ≥ 50% | Controls were > 50 years old without coronary stenosis > 30% and without history of MI, coronary artery bypass grafting, percutaneous coronary intervention, or heart transplant | 660 | 515 | [^20^](#_ENREF_12) |  |
|  |  |  |  |  |  |  |  |
| EPIC CAD | Nested case-control | The EPIC (European Prospective Study into Cancer and Nutrition) study sub-cohorts from the UK were used. Subjects were collected in collaboration with general practitioners, mainly in Cambridgeshire and Norfolk. Cases were individuals who developed fatal or non-fatal CAD during an average follow-up of 11 years ending June 2006. Participants were identified if they had a hospital admission and/or died with CAD as the underlying cause. CAD was defined as cause of death codes ICD-9 410-414 or ICD-10 I20-I25, and hospital discharge codes ICD-10 I20.0, I21, I22, or I23 according to the International Classification of Diseases, 9^th^ and 10^th^ revisions, respectively. | Controls were study participants who remained free of any cardiovascular disease during follow-up (defined as ICD-9 401-448 and ICD-10 I10-I79) | 1,386 | 7,037 | [^21^](#_ENREF_13) |  |
|  |  |  |  |  |  |  |  |
| FIA3 | Nested case-control | Cases of MI occurring in participants from Vasterbotten Intervention Program (VIP), WHO’s Multinational Monitoring of Trends and Determinants in Cardiovascular Disease (MONICA) study in northern Sweden and the Mammography Screening Project (MSP) in Vasterbotten | Individuals free of MI from VIP and MSP | 2,473 | 2,047 | ^22,23^ |  |
|  |  |  |  |  |  |  |  |
| GoDARTS CAD | Case-control | The GoDARTS (Genetics of Diabetes Audit and Research in Tayside Scotland) study is a joint initiative of the Department of Medicine and the Medicines Monitoring Unit (MEMO) at the University of Dundee, the diabetes units at three Tayside healthcare trusts (Ninewells Hospital and Medical School, Dundee; Perth Royal Infirmary; and Stracathro Hospital, Brechin), and a large group of Tayside general practitioners with an interest in diabetes care. Cases were first-ever CAD event, defined as fatal and non-fatal myocardial infarction, unstable angina, or coronary revascularization. | Controls were free of CAD, stroke, and peripheral vascular disease | 1,568 | 2,772 | [^24^](#_ENREF_16) |  |
|  |  |  |  |  |  |  |  |
| EGCUT |  | CAD or MI cases were ascertained from the Estonian Biobank (Estonian Genome Center at the University of Tartu) using the medical history and current health status that is recorded according to ICD-10 codes (CAD defined with ICD-10 I20-I25). | Controls were selected from the Estonian Biobank (Estonian Genome Center at the University of Tartu) who did not have any record of cardiovascular diseases (ICD-10 I10-I79). | 392 | 777 | [^25^](#_ENREF_17) |  |
|  |  |  |  |  |  |  |  |
| German CAD North |  | The German North cohort includes individuals from GerMIFS4, PopGen, and HNR with MI or CAD. | Controls were derived from population-based studies in Germany. | 4,464 | 2,886 | [^26-28^](#_ENREF_18) |  |
|  |  |  |  |  |  |  |  |
| German CAD South |  | The German South cohort includes samples from GerMIFS3 and Munich-MI with MI or CAD. | Controls were derived from population-based studies in Germany. | 5,255 | 2,921 | ^29,30^ |  |
|  |  |  |  |  |  |  |  |
| HUNT | Case-control | MI Cases were retrospectively identified as HUNT 2 and HUNT 3 participants diagnosed with acute MI (ICD-10 I21 or ICD-9 410) in the medical departments at the two local hospitals in Nord-Trøndelag County from December 1987 to June 2011. | Controls were selected among HUNT 2 and HUNT 3 participants with available DNA (N = 70,300) after excluding individuals with the following hospital diagnosed or self-reported conditions in themselves or known 1st and/or 2nd degree family members: MI, angina, heart failure, stroke, aortic aneurysm, atherosclerosis, intermittent claudication, and registered percutaneous coronary angioplasty procedures or bypass surgery. | 2,351 | 2,348 | [^31^](#_ENREF_23) |  |
|  |  |  |  |  |  |  |  |
| Bio*Me* Biobank | Case-control | CAD cases were ascertained from the Bio*Me* Biobank using the electronic health record with ICD9 codes 410.xx to 414.xx and abnormal stress test or abnormal coronary angiography | Controls were individuals from the Bio*Me* Biobank who did not meet the criteria for cases | 704 | 1,729 | NIH dbGaP Study Accession phs000388.v1.p1 |  |
|  |  |  |  |  |  |  |  |
| MDC | Prospective cohort | Prevalent and incident nonfatal or fatal MI | Participants free of CHD at baseline and during follow-up | 2,283 | 4,511 | [^32^](#_ENREF_24) |  |
|  |  |  |  |  |  |  |  |
| MHI | Case-control | Cases were ascertained from the Montreal Heart Institute Biobank. CAD was defined as the presence of MI, percutaneous coronary intervention, or coronary artery bypass grafting | Controls were individuals from the Montreal Heart Institute Biobank who were free of history of MI, percutaneous coronary intervention, or coronary artery bypass grafting | 3,990 | 6,585 | ^33,34^ |  |
|  |  |  |  |  |  |  |  |
| OHS | Case-control | Cases had angiographically confirmed coronary artery disease (>1 coronary artery with >50% stenosis) and did not have type 2 diabetes; ≤ 50 years old for males and ≤ 50 years old for females | Asymptomatic males > 65, females > 70 | 1,024 | 2,267 | [^35^](#_ENREF_27) |  |
|  |  |  |  |  |  |  |  |
| PAS-AMC | Case-control | Symptomatic CAD before 51 years of age, defined as MI, coronary revascularization, or evidence of at least 70% stenosis in a major epicardial coronary artery | More than 95% of the controls are from the same region as cases | 728 | 808 | [^36^](#_ENREF_28) |  |
|  |  |  |  |  |  |  |  |
| PennCath | Case-control | Cases had angiographically confirmed coronary artery disease (>1 coronary artery with 50% stenosis); ≤ 55 years old for males and ≤ 60 years old for females | Normal coronary angiography in men > 40 years old and women > 45 years old | 683 | 156 | [^37^](#_ENREF_29) |  |
|  |  |  |  |  |  |  |  |
| PROCARDIS | Case-control | Symptomatic CAD before age 66. CAD was defined as clinically documented evidence of myocardial infarction, coronary artery bypass grafting, acute coronary syndrome, coronary angioplasty, or stable angina | No personal or sibling history of CAD before age 66 | 2,490 | 2,220 | [^38^](#_ENREF_30) |  |
|  |  |  |  |  |  |  |  |
| VHS | Case-control | Documented MI, coronary artery bypass grafting, CAD (by angiography) in males ≤ 45 years old and females ≤ 50 years old | Normal coronary angiography in males > 60 years old or females > 65 years old. | 176 | 164 | [^39^](#_ENREF_31) |  |
|  |  |  |  |  |  |  |  |
| WHI | Prospective cohort | Cases were individuals from the Women’s Health Initiative who had incident MI, coronary revascularization, hospitalized angina or death due to coronary disease | Participants free of CHD on follow-up | 2,860 | 14,960 | [^40^](#_ENREF_32) |  |
|  |  |  |  |  |  |  |  |
| Discovery study total | |  |  | **42,335** | **78,240** |  |  |

ATVB: Italian Atherosclerosis, Thrombosis, and Vascular Biology Study; BHF-FHS: British Heart Foundation Family Heart Study; BioVU: Vanderbilt University Medical Center Biorepository; GoDARTS: Genetics of Diabetes Audit and Research Tayside; FIA3: First-time incidence of myocardial infarction in the AC county 3; EGCUT: Estonian Genome Centre, University of Tartu; EPIC: European Prospective Study into Cancer and Nutrition; HUNT: Nord-Trøndelag health study; IPM: Mt. Sinai Institute for Personalized Medicine Biobank; MDC: Malmo Diet and Cancer Study-Cardiovascular Cohort; MHI: Montreal Heart Institute Study; OHS: Ottawa Heart Study; PAS-AMC; Premature Atherosclerosis Study at Academic Medical Center Amsterdam; PennCath: University of Pennsylvania Catheterization Study; PROCARDIS: Precocious Coronary Artery Disease Study; VHS: Verona Heart Study; WHI: Women’s Health Initiative. MI: myocardial infarction; CAD: coronary artery disease.

**Supplemental** Table 2: **Sources of cases and controls in the replication study**

| Study (Ancestry) | Design | Case definition | Control definition | N Cases | N Controls | Reference |
| --- | --- | --- | --- | --- | --- | --- |
| BRAVE (SA) | Case-control | First-ever troponin-confirmed acute MI | Hospital controls frequency matched by age and sex | 2,971 | 2,784 | N/A |
| CCHS (EA) | Prospective cohort | Fatal and non-fatal MI and other coronary events according to ICD-10 codes I20-I25 | Participants from the CCHS cohort who were free from coronary disease at baseline and after follow-up | 2,020 | 6,087 | [^41^](#_ENREF_33) |
| CIHDS/ CGPS (EA) | Case-control | Fatal and non-fatal MI and other coronary events according to ICD-10 codes I20-I25 | Age- and sex-matched population controls free from coronary disease | 8,079 | 10,367 | [^41^](#_ENREF_33) |
| EPIC-CVD (EA) | Case-cohort | Fatal and non-fatal MI and other coronary events according to ICD-10 codes I20-I25 | A randomly-selected subcohort of participants from the EPIC cohort who were free from coronary disease at baseline and after follow-up | 3,873 | 7,914 | [^42^](#_ENREF_34) |
| MORGAM (EA) | Case-cohort | Fatal and non-fatal MI and other coronary events according to ICD-10 codes I20-I25 | A randomly-selected subcohort of participants from the MORGAM cohorts who were free from coronary disease and stroke at baseline and after follow-up | 2,153 | 2,118 | ^43,44^ |
| PROMIS (SA) | Case-control | First-ever troponin-confirmed acute MI | Hospital controls frequency matched by age and sex | 10,137 | 11,935 | [^45^](#_ENREF_37) |
| PROSPER (EA) | Nested case-control | Fatal and non-fatal MI and other coronary events according to ICD-10 codes I20-I25 | Age- and sex-matched participants from the PROSPER trial free of coronary disease at baseline and after follow-up | 641 | 638 | [^46^](#_ENREF_38) |
| WOSCOPS (EA) | Nested case-control | Fatal and non-fatal MI and other coronary events according to ICD-10 codes I20-I25 | Age-matched men from the WOSCOPS trial free of coronary disease at baseline and after follow-up | 659 | 687 | [^47^](#_ENREF_39) |
| Replication study total | |  |  | **30,533** | **42,530** |  |

EA: European Ancestry; SA: South Asian Ancestry; BRAVE: Bangladesh Risk of Acute Vascular Events Study; CCHS: Copenhagen City Heart Study; CGPS: Copenhagen General Population Study; CIHDS: Copenhagen Ischaemic Heart Disease Study; EPIC-CVD: European Prospective Investigation into Cancer and Nutrition Study; MORGAM: MOnica Risk, Genetics, Archiving and Monograph project; PROMIS: Pakistan Risk of Myocardial Infarction Study; PROSPER: Prospective Study of Pravastatin in the Elderly at Risk clinical trial; WOSCOPS: West of Scotland Coronary Prevention Study; N/A: None available.

**Supplemental Table 3: Discovery, replication and combined findings for the 28 variants that reached an association P value with CAD of < 1 x 10^-6^ in the discovery cohort**

|  |  |  |  |  |  | **Discovery** | | | | **Replication** | | | | **Meta-analysis** | |
| --- | --- | --- | --- | --- | --- | --- | --- | --- | --- | --- | --- | --- | --- | --- | --- |
| **Exome Chip ID** | **CHR** | **POS** | **Gene** | **Allele1/2** | **Allele1Freq** | **Cases/Controls** | **Odds Ratio (CI 95%)** | **P-value** | **Het P-value** | **Cases/Controls** | **OR (CI 95%)** | **P-value** | **Het P-value** | **OR (CI 95%)** | **P-value** |
| exm-rs16986953 | 2 | 19942473 | none | A/G | 0.08 | 36376/59528 | 1.11 (1.07-1.15) | 8.17x10^-08^ | 0.02 | 12253/20131 | 1.05 (1.00-1.11) | 0.06 | 0.22 | 1.09(1.06-1.12) | 5.95x10^-08^ |
| exm-rs1250229 | 2 | 216304384 | none | T/C | 0.26 | 42332/78235 | 1.08 (1.06-1.1) | 1.22x10^-12^ | 0.18 | 30531/42529 | 1.01 (0.99-1.03) | 0.38 | 0.02 | 1.05 (1.03-1.06) | 3.48x10^-09^ |
| exm-rs2943634 | 2 | 227068080 | none | A/C | 0.33 | 42332/78233 | 0.95 (0.93-0.97) | 1.57x10^-07^ | 0.02 | 30523/42511 | 0.99 (0.96-1.01) | 0.31 | 0.73 | 0.96 (0.95-0.98) | 2.85x10^-06^ |
| exm-rs2943641 | 2 | 227093745 | none | T/C | 0.36 | 42331/78228 | 0.95 (0.93-0.97) | 1.95x10^-07^ | 0.02 | 30531/42528 | 0.99 (0.97-1.01) | 0.3 | 0.38 | 0.97 (0.95-0.98) | 3.11x10^-06^ |
| exm-rs2972146 | 2 | 227100698 | none | G/T | 0.36 | 42328/78220 | 0.95 (0.93-0.97) | 2.01x10^-07^ | 0.04 | 22453/32163 | 0.99 (0.96-1.01) | 0.33 | 0.31 | 0.96 (0.95-0.98) | 1.55x10^-06^ |
| exm275853 | 2 | 233633460 | *KCNJ13,GIGYF2* | A/G | 0.35 | 42332/78229 | 1.06 (1.04-1.08) | 1.46x10^-08^ | 0.5 | 30528/42521 | 1.03 (1.01-1.06) | 0.007 | 0.88 | 1.05 (1.03-1.06) | 1.48x10^-09^ |
| exm2255118 | 2 | 233699415 | *GIGYF2* | G/A | 0.45 | 42333/78238 | 1.05 (1.03-1.07) | 9.10x10^-08^ | 0.13 | 30532/42526 | 1.02 (1.00-1.05) | 0.03 | 0.97 | 1.04 (1.03-1.06) | 4.02x10^-08^ |
| exm359878 | 3 | 153839866 | *ARHGEF26* | G/C | 0.13 | 39258/62925 | 0.92 (0.89-0.95) | 8.28x10^-09^ | 0.43 | 20394/30594 | 1 (0.96-1.04) | 0.9 | 0.74 | 0.94 (0.92-0.97) | 1.75x10^-06^ |
| exm414316 | 4 | 95496882 | *PDLIM5* | T/C | 0.31 | 42335/78234 | 0.95 (0.93-0.97) | 5.97x10^-07^ | 0.4 | 30532/42528 | 0.98 (0.95-1.00) | 0.1 | 0.83 | 0.96 (0.95-0.98) | 6.41x10^-07^ |
| exm473551 | 5 | 121413208 | *LOX* | T/C | 0.17 | 42327/78220 | 1.07 (1.04-1.09) | 1.72x10^-07^ | 0.15 | 30524/42522 | 1.02 (1.00-1.05) | 0.1 | 0.57 | 1.05 (1.03-1.07) | 5.02x10^-07^ |
| exm-rs3130683 | 6 | 31888367 | *C2* | C/T | 0.14 | 39494/72267 | 0.91 (0.88-0.94) | 7.87x10^-08^ | 0.85 | 30450/42485 | 0.91 (0.88-0.95) | 2.97x10^-05^ | 0.54 | 0.91 (0.89-0.94) | 1.04x10^-11^ |
| exm-rs1053924 | 6 | 32120715 | none | T/C | 0.30 | 39497/72302 | 0.94 (0.92-0.97) | 7.07x10^-07^ | 0.99 | 20396/30592 | 0.95 (0.92-0.98) | 0.0005 | 0.36 | 0.95 (0.93-0.96) | 1.52x10^-09^ |
| exm-rs11042937 | 11 | 10745394 | none | T/G | 0.49 | 42335/78234 | 1.05 (1.03-1.07) | 3.21x10^-08^ | 0.94 | 30533/42527 | 1.03 (1.00-1.05) | 0.02 | 0.28 | 1.04 (1.03-1.06) | 1.18x10^-08^ |
| exm-rs11172113 | 12 | 57527283 | *LRP1* | C/T | 0.41 | 42335/78234 | 1.06 (1.04-1.08) | 1.78x10^-08^ | 0.008 | 28503/36433 | 1.06 (1.03-1.08) | 1.16x10^-06^ | 0.25 | 1.06 (1.04-1.07) | 9.25x10^-14^ |
| exm2267392 | 12 | 111385296 | none | G/A | 0.50 | 39502/72328 | 0.95 (0.93-0.97) | 7.90x10^-07^ | 0.53 | 30530/42528 | 0.99 (0.96-1.01) | 0.24 | 0.36 | 0.97 (0.95-0.98) | 6.58x10^-06^ |
| exm2271796 | 12 | 112354531 | none | C/T | 0.17 | 39495/72302 | 0.94 (0.91-0.96) | 5.27x10^-07^ | 0.53 | 30510/42501 | 0.99 (0.96-1.02) | 0.4 | 0.61 | 0.96 (0.94-0.98) | 1.74x10^-05^ |
| exm1038037 | 12 | 112375990 | *TMEM116* | C/A | 0.17 | 39495/72314 | 0.93 (0.91-0.96) | 2.91x10^-07^ | 0.47 | 30526/42517 | 0.99 (0.96-1.02) | 0.42 | 0.6 | 0.96 (0.94-0.98) | 1.29x10^-05^ |
| exm-rs17696736 | 12 | 112486818 | *NAA25* | G/A | 0.45 | 39501/72325 | 1.06 (1.04-1.08) | 8.20x10^-09^ | 0.36 | 20396/30595 | 1.02 (0.99-1.05) | 0.12 | 0.78 | 1.05 (1.03-1.06) | 1.64x10^-08^ |
| exm1049349 | 12 | 124427306 | *CCDC92* | A/T | 0.32 | 42327/78211 | 0.95 (0.93-0.97) | 4.32x10^-07^ | 0.02 | 30526/42524 | 0.98 (0.96-1.01) | 0.16 | 0.1 | 0.96 (0.95-0.98) | 1.43x10^-06^ |
| exm-rs11057830 | 12 | 125307053 | *SCARB1* | A/G | 0.15 | 42331/78237 | 1.09 (1.06-1.11) | 3.69x10^-10^ | 0.2 | 20395/30592 | 1.07 (1.03-1.11) | 0.0003 | 0.58 | 1.08 (1.06-1.10) | 4.61x10^-13^ |
| exm-rs247616 | 16 | 56989590 | none | T/C | 0.32 | 42333/78214 | 0.95 (0.93-0.97) | 1.01x10^-07^ | 0.06 | 30529/42528 | 0.96 (0.93-0.99) | 0.0008 | 0.19 | 0.95 (0.94-0.97) | 4.29x10^-10^ |
| exm-rs3764261 | 16 | 56993324 | none | A/C | 0.32 | 42334/78231 | 0.95 (0.93-0.97) | 4.04x10^-08^ | 0.08 | 22451/32162 | 0.97 (0.94-0.99) | 0.06 | 0.12 | 0.95 (0.94-0.97) | 4.56x10^-09^ |
| exm-rs1800775 | 16 | 56995236 | none | A/C | 0.49 | 38810/62756 | 0.95 (0.93-0.96) | 2.21x10^-08^ | 0.13 | 22445/32148 | 0.97 (0.95-1.00) | 0.03 | 0.28 | 0.96 (0.94-0.97) | 9.83x10^-09^ |
| exm-rs2000999 | 16 | 72108093 | HPR | A/G | 0.20 | 38338/71604 | 1.07 (1.04-1.1) | 7.57x10^-08^ | 0.64 | 20393/30592 | 1.03 (1.00-1.06) | 0.07 | 0.06 | 1.05 (1.03-1.08) | 7.64x10^-08^ |
| exm1323676 | 17 | 40257163 | *DHX58* | C/T | 0.17 | 38763/61511 | 1.07 (1.04-1.1) | 5.93x10^-07^ | 0.05 | 18534/24193 | 1.02 (0.98-1.05) | 0.4 | 0.34 | 1.05 (1.03-1.07) | 4.96x10^-06^ |
| exm2272546 | 17 | 62401118 | *PECAM1* | T/C | 0.47 | 42332/78229 | 0.95 (0.93-0.97) | 1.82x10^-07^ | 0.46 | 30533/42528 | 0.98 (0.96-1.00) | 0.04 | 0.5 | 0.96 (0.95-0.98) | 1.15x10^-07^ |
| exm-rs6504218 | 17 | 62408299 | none | A/G | 0.47 | 42333/78225 | 0.95 (0.93-0.97) | 9.72x10^-08^ | 0.47 | 29890/41889 | 0.98 (0.96-1.00) | 0.07 | 0.64 | 0.96 (0.95-0.98) | 1.72x10^-07^ |
| exm1356489 | 17 | 73926121 | *FBF1* | A/C | 0.22 | 41935/77408 | 0.95 (0.92-0.97) | 9.13x10^-07^ | 0.35 | 11664/13189 | 0.97 (0.93-1.02) | 0.23 | 0.7 | 0.95 (0.93-0.97) | 9.27x10^-07^ |

Chr, Chromosome; POS, Position (HG19); Allele 1 Freq, frequency of allele 1; OR, Odds Ratio; CI, Confidence interval; Het P-value, heterogeneity P-value. A Bonferroni corrected p value of 1.7x10^-5^ f (n=29,383 SNPs) would indicate significant heterogeneity.

**Supplemental Table 4: List of variants in high LD with each of the novel CAD associated variants**

| **Locus Name** |  | **Lead Variant** | **Number of variants in high LD (r^2^>0.8)** |
| --- | --- | --- | --- |
| ***CETP*** |  | rs1800775 | 1 |
| ***SCARB1*** |  | rs11057830 | 8 |
| ***LRP1*** |  | rs11172113 | 1 |
| ***MRVI1-CTR9*** |  | rs11042937 | 7 |
| ***C2*** |  | rs3130683 | 14 |
| ***KCNJ13-GIGYF2*** | | rs1801251 | 111 |

Number of variants in high LD (r^2^ > 0.8) with each lead variant in 1000G phase 1 EUR data, identified using Haploreg V3.

**Supplemental Table 5: Notable associations between novel CAD variants and gene expression in *cis***

| **Locus** | **Transcript** | **Tissue** | **CAD associated SNP** | **P value** | **Direction** | **Best eSNP** | **P value** | **r2 (Lead variant and Best eSNP)** |
| --- | --- | --- | --- | --- | --- | --- | --- | --- |
| ***CETP*** | *CETP* | Monocytes^48^ | rs1800775 | 1.75x10^-08^ | + | rs7205804 | 5.96x10^-16^ | 0.77 |
|  | *CETP* | Monocyte^1^ | rs1800775 | 1.31x10^-07^ | + | rs7205804 | 2.08x10^-10^ | 0.77 |
|  | *CETP* | Monocytes^49^ | rs1800775 | 2.64x10^-06^ | + | rs7205804 | 2.79x10^-10^ | 0.77 |
|  | *CETP* | Liver^2^ | rs1800775 | 1.93x10^-07^ | + | rs36229491 | 1.43x10^-11^ | 0.45 |
| ***SCARB1*** | *SCARB1* | Intestine^50^ | rs11057841 | 3.88x10^-06^ | NA | rs11057841 | 3.88x10^-06^ | 0.92 |
| ***LRP1*** | *LRP1* | Omental adipose^51^ | rs11172113 | 1.55x10^-10^ | NA | rs11172113 | 1.55x10^-10^ | Same SNP |
|  | *LRP1* | Subcutaneous adipose^51^ | rs11172113 | 9.22x10^-08^ | NA | rs11172113 | 9.22x10^-08^ | Same SNP |
|  | *LRP1* | Internal mammary artery^2^ | rs11172113 | 1.59x10^-07^ | - | rs11172113 | 1.59x10^-07^ | Same SNP |
|  | *LRP1* | Atherosclerotic aortic arterial wall^2^ | rs11172113 | 8.77x10^-06^ | - | rs11172113 | 8.77x10^-06^ | Same SNP |
| ***C2*** | *CYP21A2* | Whole blood^52^ | rs3130683 | 1.41x10^-28^ | NA | rs3130287 | 1.41x10^-28^ | 0.87 |
| ***KCNJ13-GIGYF2*** | *GIGYF2* | Whole blood^53^ | rs1801251 | 8.68x10^-12^ | + | rs6717841 | 8.68x10^-12^ | 1 |
|  | *GIGYF2* | Whole blood^52^ | rs1801251 | 1.78x10^-11^ | NA | rs1801251 | 1.78x10^-11^ | Same SNP |
|  | *KCNJ13* | Intestine^50^ | rs1801251 | 1.65x10^-10^ | NA | rs6738386 | 1.65x10^-10^ | 1 |

CAD; coronary artery disease; CAD associated SNP; CAD associated lead or high LD proxy SNP with an eQTL association; SNP; single nucleotide polymorphism; Direction, + indicates higher expression with the CAD risk allele; NA, not available; Best eSNP; SNP at the locus with highest P value for eQTL association.

**Supplemental Table 6: Association P-values of CAD variants with selected cardiovascular risk factors**

| Locus | Locus Name | Lead Variant | LDL | HDL | TG | SBP | DBP | PP | BMI | T2D | Smoking |
| --- | --- | --- | --- | --- | --- | --- | --- | --- | --- | --- | --- |
| New Loci |  |  |  |  |  |  |  |  |  |  |  |
| 2q37 | *KCNJ13-GIGYF2* | rs1801251 | 0.13 | 0.7 | 0.9 | 0.88 | 0.66 | 0.81 | 0.27 | 0.05 | 0.69 |
| 6p21 | *C2* | rs3130683 | 0.001 | 0.29 | 1.2x10^-4^ | 0.21 | 0.93 | 0.04 | 0.21 | **2.7x10^-5^** | 0.38 |
| 11p15 | *MRVI1-CTR9* | rs11042937 | 0.62 | 0.24 | 0.48 | 0.004 | 0.01 | 0.03 | 0.16 | 0.98 | 0.73 |
| 12q13 | *LRP1* | rs11172113 | 0.53 | 0.05 | 0.26 | 0.22 | 3 x10^-4^ | 0.29 | 0.17 | 0.64 | 0.44 |
| 12q24 | *SCARB1* | rs11057830 | **2.6x10^-5^** | 0.002 | **8.3x10^-5^** | 0.06 | 0.07 | 0.36 | 0.41 | 0.76 | 0.18 |
| 16q13 | *CETP* | rs1800775 | **8.5x10^-24^** | **3.3x10^-644^** | **1.3x10^-26^** | 0.71 | 0.24 | 0.37 | 0.16 | 0.69 | 0.89 |
| Known Loci |  |  |  |  |  |  |  |  |  |  |  |
| 1p32 | *PCSK9* | rs11206510 | **2.4x10^-57^** | 0.16 | 0.001 | 0.86 | 0.28 | 0.45 | 0.07 | 0.29 | 0.78 |
| 1p32 | *PPAP2B* | rs17114036 | 0.6 | 0.18 | 0.19 | 0.40 | 0.72 | 0.03 | 0.03 | 0.40 | 0.18 |
| 1p13 | *SORT1* | rs602633 | **1.5x10^-261^** | **3.5x10^-14^** | 0.003 | 0.93 | 0.73 | 0.72 | 0.06 | 0.03 | 0.73 |
| 1q21 | *IL6R* | rs4845625 | 0.87 | 0.95 | 0.53 | 0.27 | 0.20 | 0.90 | 0.09 | 0.63 | 0.08 |
| 1q41 | *MIA3* | rs17465637 | 0.62 | 0.78 | 0.86 | 0.59^d^ | 0.98^d^ | 0.21^d^ | 0.59 | 3.5x10^-4^ | 0.65 |
| 2p24 | *APOB* | rs515135 | **1.1x10^-178^** | 0.009 | 1.4x10^-4^ | 0.64 | 0.10 | 0.23 | 0.08 | 0.74 | 0.13 |
| 2p21 | *ABCG5-ABCG8* | rs6544713 | **4.8x10^-83^** | 0.16 | 0.001 | 0.90 | 0.90 | 0.69 | 0.03 | 0.50 | 1.00 |
| 2p11 | *VAMP5-VAMP8-GGCX* | rs1561198 | 0.02 | 0.36 | 0.13 | 0.98 | 0.95 | 0.02 | 0.02 | 0.72 | 0.75 |
| 2q22 | *ZEB2* | rs2252641 | 0.49 | 0.15 | 0.21 | 0.02 | 0.03 | 0.89 | 0.12 | 0.05 | 0.52 |
| 2q33 | *WDR12* | rs6725887 | **1.3x10^-5^** | 0.1 | 0.04 | 0.94 | 0.09 | 0.01 | 0.19 | 0.00 | 0.54 |
| 3q22 | *MRAS* | rs9818870 | 0.31 | 0.51 | 0.85 | 0.04 | 0.02 | 0.001 | 0.06 | 0.12 | 0.76 |
| 4q12 | *REST-NOA1* | rs17087335 | 0.31 | 0.66 | 0.37 | 0.58 | 0.51 | 0.09 | 0.22 | 0.40 | 0.38 |
| 4q31 | *EDNRA* | rs1878406 | 0.94 | 0.32 | 0.52 | 0.09 | 0.81 | 0.01 | 0.65 | 0.82 | 0.79 |
| 4q32 | *GUCY1A3* | rs7692387 | 0.68 | 0.85 | 0.19 | 0.01 | **3.4x10^-5^** | 0.75 | 0.55 | 0.04 | 0.10 |
| 5q31 | *SLC22A4-SLC22A5* | rs273909 | **2.3x10^-5^** | 0.03 | 0.35 | 0.88 | 0.47 | 0.38 | 0.78 | 0.88 | 0.80 |
| 6p21 | *ANKS1A* | rs17609940 | 0.72 | 0.003 | 0.02 | 0.25 | 0.14 | 0.85 | 0.02 | 0.24 | 0.73 |
| 6p21 | *KCNK5* | rs10947789 | 0.28 | 0.22 | 0.68 | 0.62 | 0.02 | 0.34 | 0.23 | 0.99 | 0.79 |
| 6p24 | *PHACTR1* | rs9369640 | 0.18 | 0.16 | 0.12 | 0.02 | 0.02 | 0.06 | 0.03 | 0.92 | 0.95 |
| 6q23 | *TCF21* | rs12190287 | 0.1 | 0.14 | 0.19 | 0.85 | 0.26 | 0.68 | 0.72 | 0.69 | 0.55 |
| 6q25 | *LPA* | rs3798220 | **6.1x10^-11^** | 0.84 | 0.69 | 0.14 | 0.61 | 0.10 | 0.17 | - | 0.93^b^ |
|  |  | rs2048327 | **1.3x10^-6^** | 0.64 | 0.009 | 0.47 | 0.49 | 0.05 | 0.001 | 0.58 | 0.35 |
| 6q26 | *PLG* | rs4252120 | 0.04 | 0.04 | 0.79 | 0.73 | 0.73 | 0.54 | 0.25 | 0.92 | 0.72 |
| 7p21 | *HDAC9* | rs2023938 | 0.36 | 0.8 | 0.11 | 0.01 | 0.73 | 0.004 | 0.55 | 0.74 | 0.53 |
| 7q22 | 7q22 | rs10953541 | 0.03 | 0.03 | 0.32 | 0.72 | 0.54 | 0.43 | 0.44 | 0.27 | 0.86 |
| 7q32 | *ZC3HC1* | rs11556924 | 0.09 | **1.3x10^-5^** | 0.002 | 1.3 x10^-4^ | **1.79x10^-5^** | 0.18 | 0.008 | 0.14 | 0.66 |
| 7q36 | *NOS3* | rs3918226 | 0.2 | 0.28 | 0.32 | **1.1x10^-6^** | **2.2x10^-9^** | 0.28 | 0.47 | - | - |
| 8p21 | *LPL* | rs264 | 0.09 | **8.3x10^-77^** | **2.4x10^-84^** | 0.98 | 0.79 | 0.97 | 0.11 | 0.01 | 0.42 |
| 8q24 | *TRIB1* | rs2954029 | **2.1x10^-50^** | **2.7x10^-29^** | **1x10^-107^** | 0.38 | 0.75 | 0.20 | 0.02 | 0.72 | 0.33 |
| 9p21 | *CDKN2BAS1* | rs4977574 | 0.09 | 0.14 | 0.99 | 0.73 | 0.18 | 0.51 | 0.63 | 0.04 | 0.50 |
|  |  | rs3217992 | 0.8 | 0.58 | 0.63 | 0.58 | 0.07 | 0.78 | 0.53 | 0.01 | 0.31 |
| 9q34 | *ABO* | rs579459 | **2.4x10^-44^** | 0.08 | 0.01 | 0.19 | 0.001 | 0.25 | 0.84 | 0.16 | 0.64 |
| 10p11 | *KIAA1462* | rs2505083 | 0.48 | 0.79 | 0.38 | 0.39 | 0.17 | 0.02 | 0.99 | 0.28 | 0.57 |
| 10q11 | *CXCL12* | rs501120 | 0.28 | 0.81 | 0.9 | 0.54 | 0.14 | 0.46 | 0.67 | 0.74 | 0.98 |
|  |  | rs2047009 | 0.7 | 0.36 | 0.47 | 0.67 | 0.02 | 0.12 | 0.54 | 0.50 | 0.88 |
| 10q23 | *LIPA* | rs2246833 | 0.13 | 0.23 | 0.12 | 0.39 | 0.61 | 0.48 | 0.24 | 0.48 | 0.86 |
|  |  | rs11203042 | 0.04 | 0.89 | 0.003 | 0.69 | 0.57 | 0.39 | 0.98 | 0.61 | 0.39 |
| 10q24 | *CYP17A1-CNNM2-NT5C2* | rs12413409 | 0.45 | 0.67 | 0.26 | **2x10^-9^** | **8.8x10^-6^** | **5.7x10^-8^** | **2.2x10^-8^** | 0.81 | 0.64 |
| 11p15 | *SWAP70* | rs10840293 | 0.02^g^ | 0.36^g^ | 0.65^g^ | 3.2x10^-4g^ | 3.7x10^-4g^ | 0.26^g^ | 0.31^g^ | 0.3^g^ | 0.41^g^ |
| 11q22 | *PDGFD* | rs974819 | 0.41 | 0.36 | 0.37 | 0.99 | 0.76 | 0.69 | 0.5 | 0.41 | 0.73 |
| 11q23 | *ZNF259-APOA5-APOA1* | rs964184 | **2x10^-26^** | **6x10^-48^** | **6.6x10^-224^** | 0.24 | 0.96 | 0.10 | 0.9 | 0.15 | 0.63 |
| 12q24 | *SH2B3* | rs3184504 | **4.2x10^-12^** | **4.1x10^-12^** | 0.03 | **1.7x10^-9^** | **2.3x10^-14^** | 0.11 | **9.4x10^-6^** | 0.63 | 0.01 |
| 12q24 | *KSR2* | rs11830157 | 0.52 | 0.43 | 0.26 | 0.31 | 0.37 | 0.24 | 0.27 | 0.33 | 0.63 |
| 13q12 | *FLT1* | rs9319428 | 0.38 | 0.63 | 0.73 | 0.84 | 0.11 | 0.39 | 0.001 | 0.62 | 0.89 |
| 13q34 | *COL4A1-COL4A2* | rs4773144 | 0.001 | 0.96 | 0.84 | 0.52 | 0.69 | 0.41 | 0.16 | 0.54 | 0.43 |
|  |  | rs9515203 | 0.05 | 0.28 | 0.03 | 0.001 | 0.01 | 0.04 | 0.18 | 0.02 | 0.53 |
| 14q32 | *HHIPL1* | rs2895811 | 0.42 | 0.36 | 0.54 | 0.18 | 0.76 | 0.31 | 0.83 | 0.08 | 0.54 |
| 15q22 | *SMAD3* | rs56062135 | 0.93^f^ | 0.61^f^ | 0.29^f^ | 0.15^f^ | 0.37^f^ | 0.87^f^ | 0.13^f^ | 0.46^f^ | 0.73^f^ |
| 15q25 | *ADAMTS7* | rs7173743 | 0.34 | 0.46 | 0.99 | 0.04 | 0.92 | 0.002 | 0.10 | 0.02 | 0.02 |
|  |  | rs3825807 | 0.99 | 0.49 | 0.51 | 0.69 | 0.25 | 0.1 | 0.12 | 0.003 | 1.3x10^-4^ |
| 15q26 | *MFGE8-ABHD2* | rs8042271 | 0.71^c^ | 0.75^c^ | 0.95^c^ | 0.54^c^ | 0.95^c^ | 0.23^c^ | 0.41^c^ | 0.08^c^ | 0.93^c^ |
| 15q26 | *FURIN-FES* | rs17514846 | 0.39 | 0.07 | 0.02 | **1.2x10^-5^** | 0.004 | 8.5x10^-5^ | 0.13 | 0.40 | 0.90 |
| 17p13 | *SMG6* | rs2281727 | 0.08 | 0.03 | 0.37 | 0.39 | 0.68 | 0.16 | **3.6x10^-6^** | 0.69 | 0.87 |
| 17p11 | *RAI1-PEMT-RASD1* | rs12936587 | 0.15 | 0.35 | 0.03 | 0.87 | 0.61 | 0.64 | 0.004 | 0.44 | 0.44 |
| 17q21 | *UBE2Z* | rs15563 | 0.03 | 0.16 | 0.36 | 0.72 | 0.14 | 0.14 | 0.68 | 4.8x10^-4^ | 0.27 |
| 17q23 | *BCAS3* | rs7212798 | 0.83^a^ | 0.23^a^ | 0.26^a^ | 0.03^a^ | 0.13^a^ | 0.02^a^ | 0.83^a^ | 0.55^a^ | 0.17^a^ |
| 18q21 | *PMAIP1-MC4R* | rs663129 | 0.61 | **5.5x10^-9^** | 0.001 | 0.58 | 0.91 | 0.07 | **8.8x10^-53^** | 0.002 | 0.25 |
| 19p13 | *LDLR* | rs1122608 | **8.5x10^-57^** | 0.16 | 0.53 | 0.95 | 0.66 | 0.88 | 0.02 | 0.86 | 0.46 |
| 19q13 | *ZNF507-LOC400684* | rs12976411 | 0.31 | 0.17 | 0.39 | 0.75 | 0.89 | 0.77 | 0.26 | 0.01 | 0.97 |
| 19q13 | *APOE-APOC1* | rs2075650 | **1.7x10^-214^** | **9.7x10^-26^** | **2.3x10^-21^** | 0.49 | 0.95 | 0.69 | **1.25x10^-8^** | 0.003 | 0.39 |
|  |  | rs445925 | **6.6x10^-397^** | **1.9x10^-10^** | **3.6x10^-39^** | - | - | - | 0.08 | - | 0.80 |
| 21q22 | *KCNE2* | rs9982601 | 0.04 | 0.06 | 0.49 | 0.11 | 0.26 | 0.46 | 0.36 | 0.46 | 0.55 |
| 22q11 | *POM121L9P-ADORA2A* | rs180803 | 0.76^e^ | 0.84^e^ | 0.58^e^ | 0.56^e^ | 0.64^e^ | 0.57^e^ | - | 0.53^e^ | 0.02^e^ |
|  |  |  |  |  |  |  |  |  |  |  |  |

LDL, plasma LDL-cholesterol level; TG, plasma triglyceride level; HDL, plasma HDL-cholesterol level; T2D, type-2 diabetes; SBP, systolic blood pressure DBP, diastolic blood pressure; PP pulse pressure (defined as systolic pressure minus diastolic pressure); BMI, body mass index; T2D, type-2 diabetes.

A Bonferroni corrected p value of 8.32x10^-5^ f (n=600 tests) was applied to denote significance. P-values lower than this are shown in bold.

^a^ rs7213603 was used as a proxy for rs7212798, *r*^2^=0.94. ^b^ rs9457925 was used as a proxy for rs3798220 *r*^2^=1. ^c^ rs7164299 was used as a proxy for rs8042271, *r*^2^=0.64. ^d^ rs17011681 was used as a proxy for rs17465637 *r*^2^=1.^e^ rs5760293 was used as a proxy for rs180803 *r*^2^=0.57. ^f^ rs17293632 was used as a proxy for rs56062135 *r*^2^=0.94. ^g^ rs360156 was used as a proxy for rs10840293 *r*^2^=0.96.

The maximum sample size available for each trait are: HDL-cholesterol level, 187,167; LDL-cholesterol level, 173,082; TG level, 177,860; SBP, 69,899; DBP, 69,909; PP, 74,079; BMI, 339,224; T2D cases 34,840 and controls 114,981; Smoking, 41,150.

**Supplemental Figure 1**: **Forest plots of the six novel CAD loci**

^
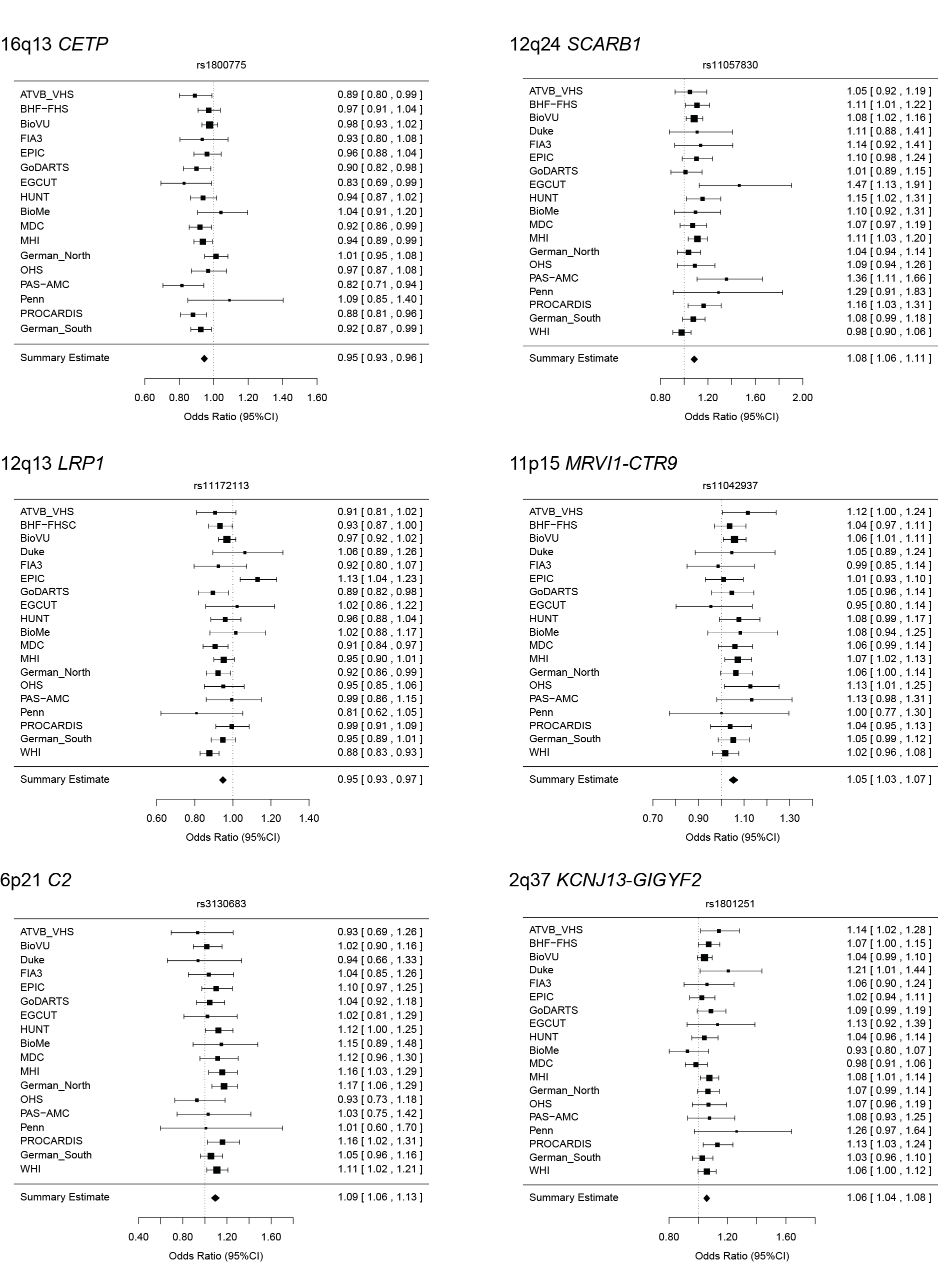
^

Forest plots for association of each of the novel loci with coronary artery disease in the discovery studies. Plots illustrate effect size (Odds ratio, log scale) and 95% confidence intervals (CIs) observed in each contributing study.

**Supplemental Figure 2**: **Regional association plots for the six novel CAD loci**

^
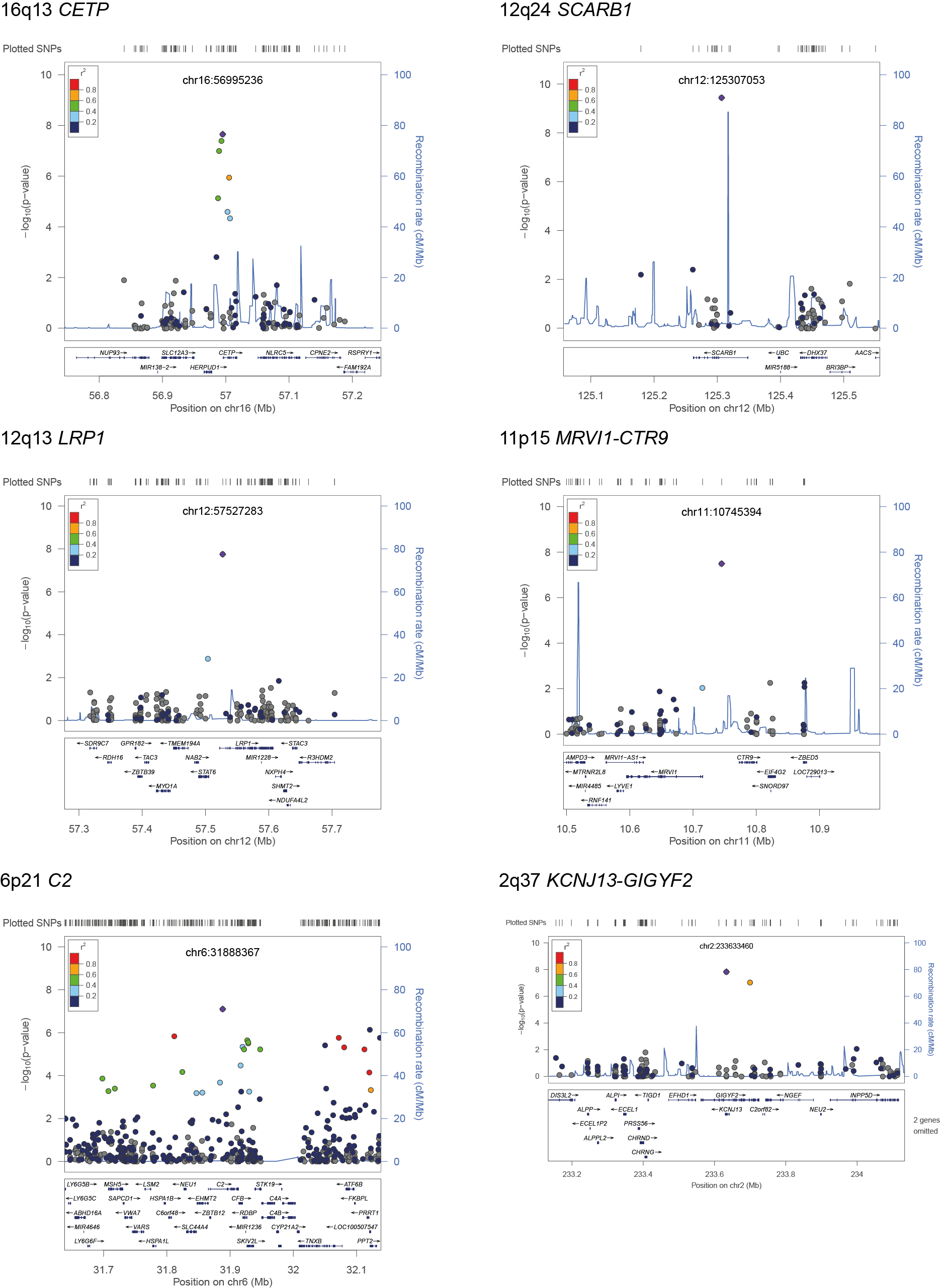
^

Regional association plots are shown for each of the novel loci in the discovery analysis. Regional plots show SNPs plotted by their positions on chromosomes against –log10 p-value for their association with CAD. The top SNP in each region is highlighted in purple. The SNPs surrounding each top SNP are color coded to reflect their LD with this variant LD (r^2^) calculations were based on the 1000 Genomes March 2012 release (EUR). Genomic coordinates refer to the hg19 sequence assembly. Plots were generated using Locus Zoom.

**Supplemental Figure 3. Functional annotation of CAD associated SNPs at the six novel loci.**


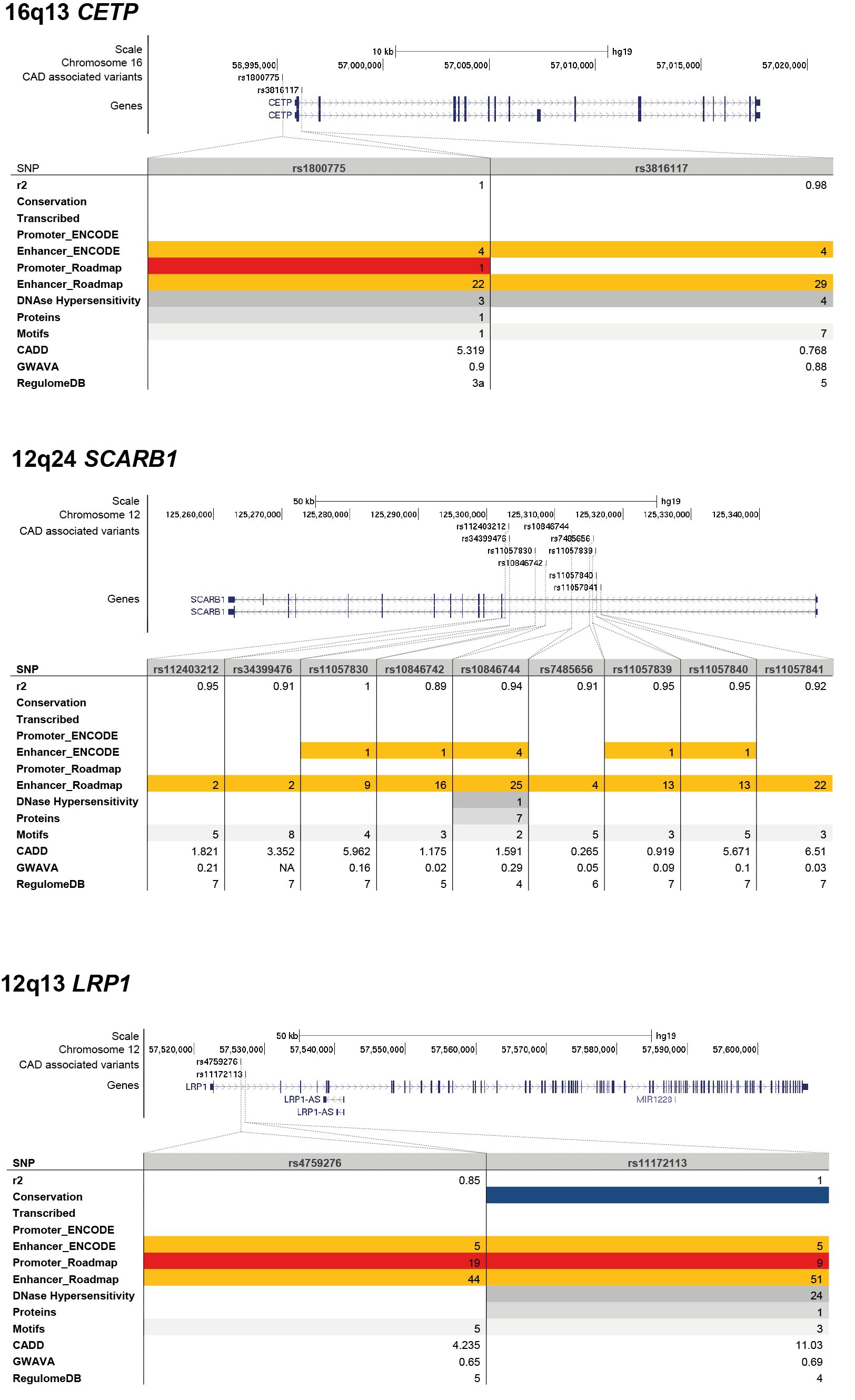


**
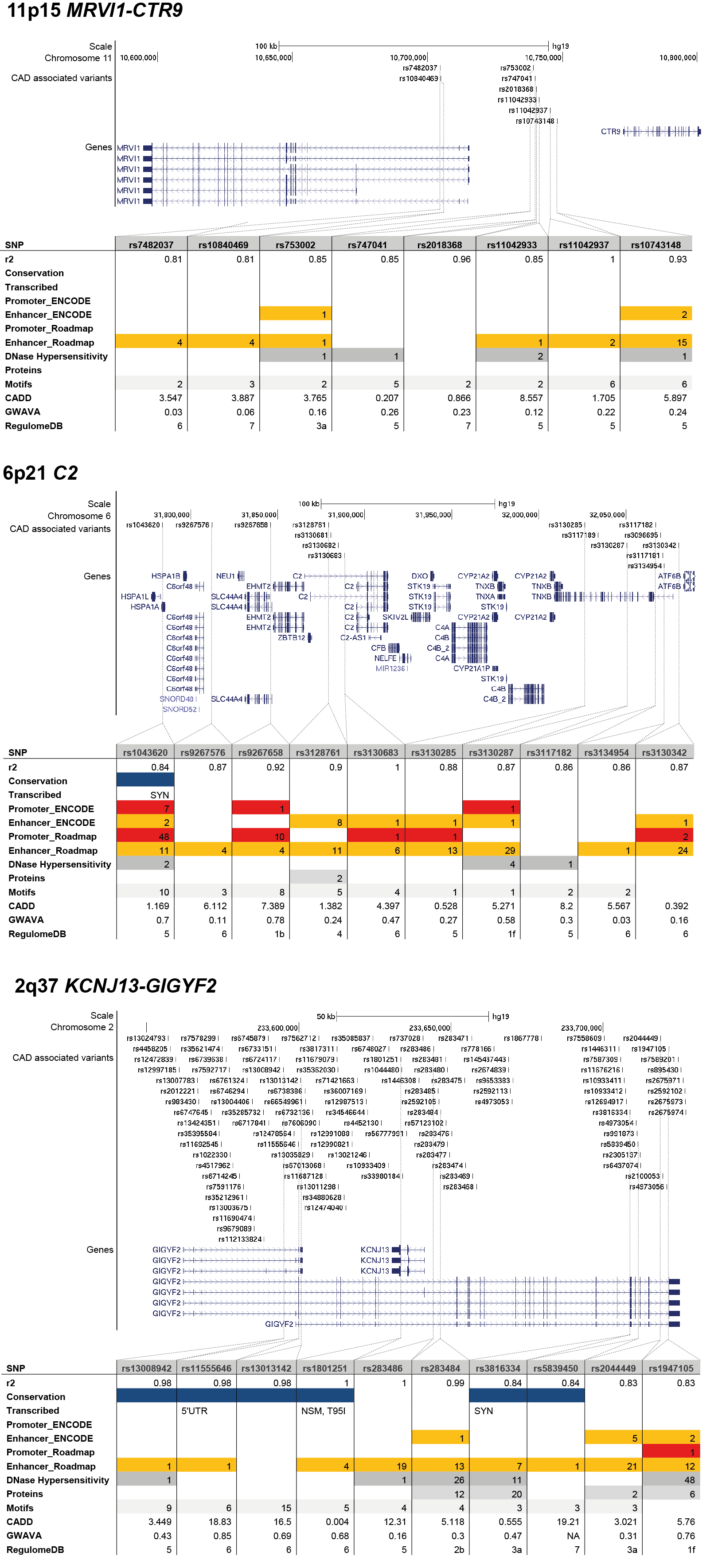
**

Each figure shows a UCSC genome browser (hg19) snapshot of the CAD associated variants as well as nearby genes. Regulatory annotation generated using Haploreg V3 (see Methods), is shown in tabular form and summarizes the regulatory features overlapping each CAD associated variant including promoter and enhancers based on chromatin state segmentation from ENCODE and Roadmap epigenome, DNase hypersensitivity, bound proteins and predicted disruption of transcription factor binding motifs. The numbers in each block represent the number of observations of each feature. Functional prediction scores for each variant from CADD, GWAVA and RegulomeDB are also shown in the lower three rows of each table. Due to the high number of variants at the 2q37 and 6p21 loci only a selection of variants are included.

**Supplemental Figure 4. Comparison of magnitude of associations with risk factor and CAD for selected variants**

**
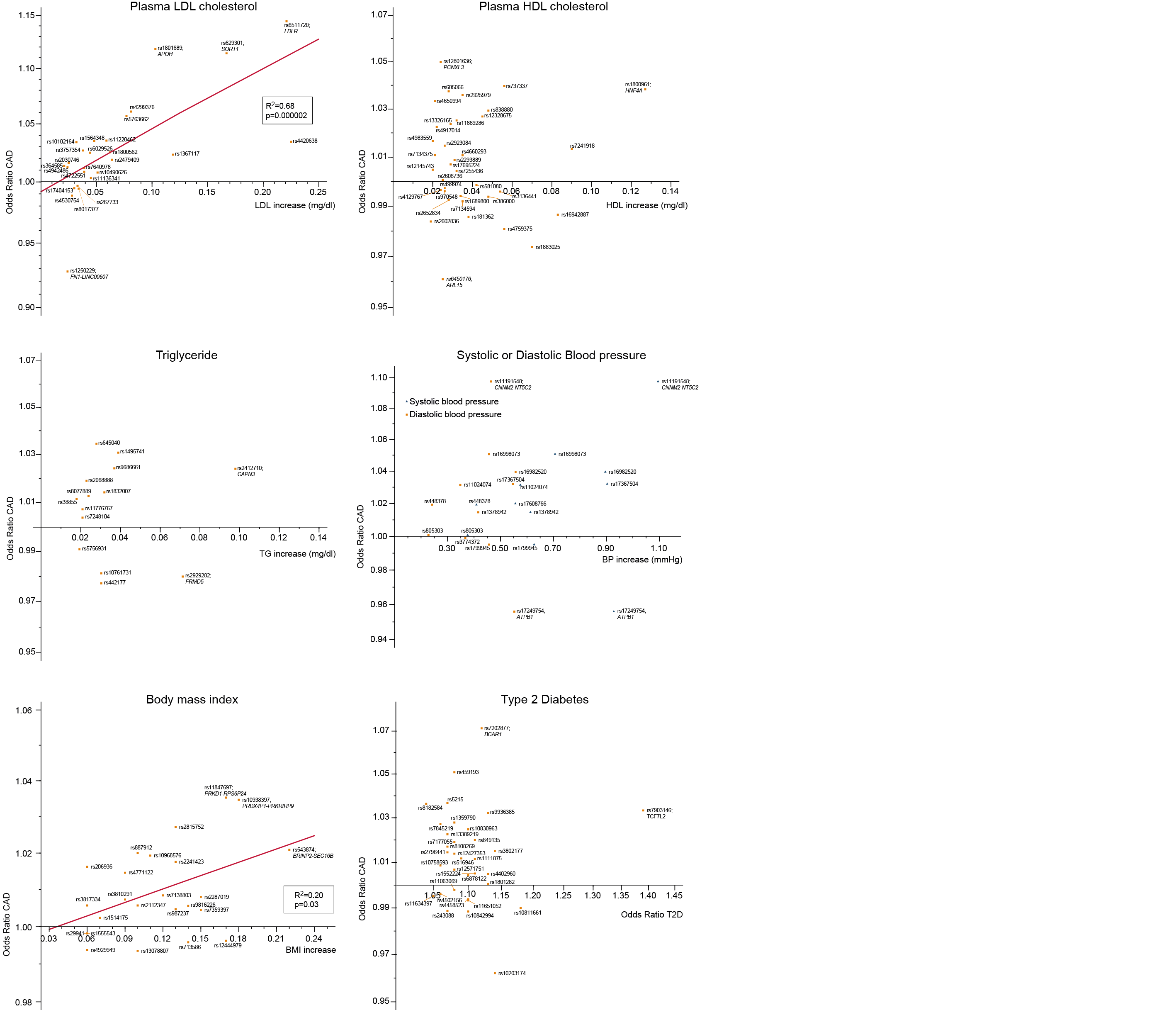
**

For variants (or proxies) available on the exome array that have a genome-wide significant association with a traditional risk factor the relative magnitudes of the reported association with the risk factor and the observed association with CAD in our data are plotted. For only two risk factors (LDL-cholesterol and BMI) was there a significant correlation.

**Supplemental References**

1. Rotival M, Zeller T, Wild PS, et al. Integrating genome-wide genetic variations and monocyte expression data reveals trans-regulated gene modules in humans. PLoS genet 2011;7:e1002367.

2. Franzén O, Ermel R, Cohain A, et al. Cardiometabolic risk loci share downstream cis- and trans-gene regulation across tissues and diseases. Science 2016;353:827-830.

3. Eicher JD, Landowski C, Stackhouse B, et al. GRASP v2.0: an update on the Genome-Wide Repository of Associations between SNPs and phenotypes. Nucleic Acids Res 2015;43:D799-804.

4. Ward LD, Kellis M. HaploReg: a resource for exploring chromatin states, conservation, and regulatory motif alterations within sets of genetically linked variants. Nucleic Acids Res. 2012; 40:D930-4.

5. Kumar P, Henikoff S, Ng PC. Predicting the effects of coding non-synonymous variants on protein function using the SIFT algorithm. Nat Protoc 2009;4:1073-81.

6. Adzhubei IA, Schmidt S, Peshkin L, et al. A method and server for predicting damaging missense mutations. Nat Methods 2010;7:248-9.

7. Davydov EV, Goode DL, Sirota M, et al. Identifying a high fraction of the human genome to be under selective constraint using GERP++. PLoS Comput Biol 2010;6:e1001025.

8. Garber M, Guttman M, Clamp M, et al. Identifying novel constrained elements by exploiting biased substitution patterns. Bioinformatics 2009;25:54-62.

9. ENCODE Project Consortium. An integrated encyclopedia of DNA elements in the human genome. Nature 2012;489:57-74.

10. Skipper M, Eccleston A, Gray N, et al. Presenting the epigenome roadmap. Nature 2015;518:313.

11. Matys V, Fricke E, Geffers R, et al. TRANSFAC: transcriptional regulation, from patterns to profiles. Nucleic Acids Res 2003;31:374–8.

12. Portales-Casamar E, Thongjuea S, Kwon AT, et al. JASPAR 2010: the greatly expanded open-access database of transcription factor binding profiles. Nucleic Acids Res 2010;38:D105–10.

13. Kircher M, Witten DM, Jain P, et al. A general framework for estimating the relative pathogenicity of human genetic variants. Nat Genet 2014;46:310-5.

14. Ritchie GR, Dunham I, Zeggini E, et al. Functional annotation of noncoding sequence variants. Nat Methods 2014;11:294-6.

15. Boyle AP, Hong EL, Hariharan M, et al. Annotation of functional variation in personal genomes using RegulomeDB. Genome research. 2012;22:1790-7.

16. Atherosclerosis, Thrombosis, and Vascular Biology Italian Study Group. No evidence of association between prothrombotic gene polymorphisms and the development of acute myocardial infarction at a young age. Circulation 2003;107:1117-22.

17. Consortium CAD, Deloukas P, Kanoni S, et al. Large-scale association analysis identifies new risk loci for coronary artery disease. Nat Genet 2013;45:25-33.

18. Samani NJ, Erdmann J, Hall AS, et al. Genomewide association analysis of coronary artery disease. N Engl J Med 2007;357:443-53.

19. Weeke P, Denny JC, Basterache L, et al. Examining Rare and Low-Frequency Genetic Variants Previously Associated with Lone or Familial Forms of Atrial Fibrillation in an Electronic Medical Record System: A Cautionary Note. Circ Cardiovasc Genet 2014.

20. Davies RW, Wells GA, Stewart AF, et al. A genome-wide association study for coronary artery disease identifies a novel susceptibility locus in the major histocompatibility complex. Circ Cardiovasc Genet 2012;5:217-25.

21. Day N, Oakes S, Luben R, et al. EPIC-Norfolk: study design and characteristics of the cohort. European Prospective Investigation of Cancer. British journal of cancer 1999;80 Suppl 1:95-103.

22. Norberg M, Blomstedt Y, Lonnberg G, et al. Community participation and sustainability--evidence over 25 years in the Vasterbotten Intervention Programme. Global health action 2012;5:1-9.

23. Stegmayr B, Lundberg V, Asplund K. The events registration and survey procedures in the Northern Sweden MONICA Project. Scandinavian journal of public health Supplement 2003;61:9-17.

24. Morris AD, Boyle DI, MacAlpine R, et al. The diabetes audit and research in Tayside Scotland (DARTS) study: electronic record linkage to create a diabetes register. DARTS/MEMO Collaboration. Bmj 1997;315:524-8.

25. Leitsalu L, Haller T, Esko T, et al. Cohort Profile: Estonian Biobank of the Estonian Genome Center, University of Tartu. International journal of epidemiology 2014.

26. Erdmann J, Stark K, Esslinger UB, et al. Dysfunctional nitric oxide signalling increases risk of myocardial infarction. Nature 2013;504:432-6.

27. Krawczak M, Nikolaus S, von Eberstein H, Croucher PJ, El Mokhtari NE, Schreiber S. PopGen: population-based recruitment of patients and controls for the analysis of complex genotype-phenotype relationships. Community genetics 2006;9:55-61.

28. Schmermund A, Möhlenkamp S, Stang A, et al. Assessment of clinically silent atherosclerotic disease and established and novel risk factors for predicting myocardial infarction and cardiac death in healthy middle-aged subjects: rationale and design of the Heinz Nixdorf RECALL Study. Risk Factors, Evaluation of Coronary Calcium and Lifestyle. American heart journal 2002;144:212-8.

29. Erdmann J, Willenborg C, Nahrstaedt J, et al. Genome-wide association study identifies a new locus for coronary artery disease on chromosome 10p11.23. Eur Heart J 2011;32:158-68.

30. Koch W, Turk S, Erl A, et al. The chromosome 9p21 region and myocardial infarction in a European population. Atherosclerosis 2011;217:220-6.

31. Krokstad S, Langhammer A, Hveem K, et al. Cohort Profile: the HUNT Study, Norway. International journal of epidemiology 2013;42:968-77.

32. Kathiresan S, Melander O, Anevski D, et al. Polymorphisms associated with cholesterol and risk of cardiovascular events. N Engl J Med 2008;358:1240-9.

33. Auer PL, Teumer A, Schick U, et al. Rare and low-frequency coding variants in CXCR2 and other genes are associated with hematological traits. Nat Genet 2014;46:629-34.

34. Dube MP, Zetler R, Barhdadi A, et al. CKM and LILRB5 Are Associated With Serum Levels of Creatine Kinase. Circ Cardiovasc Genet 2014;7:880-6.

35. McPherson R, Pertsemlidis A, Kavaslar N, et al. A common allele on chromosome 9 associated with coronary heart disease. Science 2007;316:1488-91.

36. Trip MD, Smulders YM, Wegman JJ, et al. Frequent mutation in the ABCC6 gene (R1141X) is associated with a strong increase in the prevalence of coronary artery disease. Circulation 2002;106:773-5.

37. Reilly MP, Li M, He J, et al. Identification of ADAMTS7 as a novel locus for coronary atherosclerosis and association of ABO with myocardial infarction in the presence of coronary atherosclerosis: two genome-wide association studies. Lancet 2011;377:383-92.

38. Clarke R, Peden JF, Hopewell JC, et al. Genetic variants associated with Lp(a) lipoprotein level and coronary disease. N Engl J Med 2009;361:2518-28.

39. Kathiresan S, Voight BF, Purcell S, et al. Genome-wide association of early-onset myocardial infarction with single nucleotide polymorphisms and copy number variants. Nat Genet 2009;41:334-41.

40. Women's Health Initiative Study Group. Design of the Women's Health Initiative clinical trial and observational study. The Women's Health Initiative Study Group. Controlled clinical trials 1998;19:61-109.

41. Nordestgaard BG, Benn M, Schnohr P, Tybjaerg-Hansen A. Nonfasting triglycerides and risk of myocardial infarction, ischemic heart disease, and death in men and women. JAMA 2007;298:299-308.

42. Danesh J, Saracci R, Berglund G, et al. EPIC-Heart: the cardiovascular component of a prospective study of nutritional, lifestyle and biological factors in 520,000 middle-aged participants from 10 European countries. European journal of epidemiology 2007;22:129-41.

43. Evans A, Salomaa V, Kulathinal S, et al. MORGAM (an international pooling of cardiovascular cohorts). International journal of epidemiology 2005;34:21-7.

44. Kulathinal S, Karvanen J, Saarela O, Kuulasmaa K. Case-cohort design in practice - experiences from the MORGAM Project. Epidemiologic perspectives & innovations : EP+I 2007;4:15.

45. Saleheen D, Zaidi M, Rasheed A, et al. The Pakistan Risk of Myocardial Infarction Study: a resource for the study of genetic, lifestyle and other determinants of myocardial infarction in South Asia. European journal of epidemiology 2009;24:329-38.

46. Shepherd J, Blauw GJ, Murphy MB, et al. Pravastatin in elderly individuals at risk of vascular disease (PROSPER): a randomised controlled trial. Lancet 2002;360:1623-30.

47. Shepherd J, Cobbe SM, Ford I, et al. Prevention of coronary heart disease with pravastatin in men with hypercholesterolemia. West of Scotland Coronary Prevention Study Group. N Engl J Med 1995;333:1301-7.

48. Fairfax BP, Humburg P, Makino S, et al. Innate immune activity conditions the effect of regulatory variants upon monocyte gene expression. Science. 2014;343(6175):1246949.

49. Fairfax BP, Makino S, Radhakrishnan J, et al. Genetics of gene expression in primary immune cells identifies cell type-specific master regulators and roles of HLA alleles. Nat Genet. 2012;44(5):502-10.

50. Kabakchiev B, Silverberg MS. Expression quantitative trait loci analysis identifies associations between genotype and gene expression in human intestine. Gastroenterology. 2013; 144(7):1488-96.

51. Greenawalt DM, Dobrin R, Chudin E, et al. A survey of the genetics of stomach, liver, and adipose gene expression from a morbidly obese cohort. Genome Res. 2011; 21(7):1008-16.

52. Battle A, Mostafavi S, Zhu X, et al. Characterizing the genetic basis of transcriptome diversity through RNA-sequencing of 922 individuals. Genome Res. 2014;24(1):14-24.

53. Westra HJ, Peters MJ, Esko T, et al. Systematic identification of trans eQTLs as putative drivers of known disease associations. Nat Genet. 2013; 45(10):1238-43.
